# From dysbiosis to tumorigenesis: microbiome-derived metabolites as emerging cancer biomarkers

**DOI:** 10.3389/fmicb.2026.1843755

**Published:** 2026-06-17

**Authors:** Anbalagan Sanjana, Pragasam Viswanathan

**Affiliations:** Renal Research Lab, Pearl Research Park, School of BioSciences and Technology, Vellore Institute of Technology (VIT), Vellore, Tamil Nadu, India

**Keywords:** cancer biomarkers, cancer metabolomics, dysbiosis, gut microbiome, microbial metabolites, microbiome-host interactions, multi-omics integration, tumor microenvironment

## Abstract

The gut microbiome constitutes a complex and metabolically active ecosystem that exerts profound effects on host physiology through the production of diverse small-molecule metabolites. Increasing evidence indicates that microbiome-derived metabolites function as a critical bridge linking microbial dysbiosis with tumor initiation, progression, immune modulation, and therapeutic responsiveness. Alterations in microbial metabolic outputs, including short-chain fatty acids, secondary bile acids, polyamines, indole derivatives, and other bioactive compounds, can influence epithelial barrier integrity, inflammatory signaling, epigenetic regulation, and genomic stability within the tumor microenvironment. These metabolites serve as functional intermediaries in host-microbe communication and may contribute to systemic metabolic and immunological changes that support tumorigenesis across multiple malignancies. Notably, many microbiome-associated metabolites are detectable in accessible biological matrices such as stool, blood, and urine, highlighting their potential utility as minimally invasive biomarkers for cancer risk screening, diagnosis, and prognosis. Recent advances in high-throughput sequencing, metabolomics, and multi-omics integration have enabled comprehensive characterization of microbial metabolic networks associated with cancer development. In this review, we synthesize current insights into the functional diversity of microbiome-derived metabolites and their mechanistic roles in tumor biology. We further examine analytical platforms used for metabolite profiling and discuss emerging strategies for microbiome-targeted therapeutic modulation. Finally, we outline current methodological challenges and research priorities necessary for translating microbiome-metabolite interactions into clinically actionable frameworks for precision oncology.

## 1 Introduction

The human gastrointestinal tract harbors a complex and metabolically active microbial ecosystem that plays a crucial role in maintaining host homeostasis (Qin et al., 2010; Furusawa et al., 2013). Microbiome-derived metabolites, including short-chain fatty acids, secondary bile acids, and indole derivatives, act as key mediators of host-microbe communication (Devkota et al., 2012; Furusawa et al., 2013; Pleguezuelos-Manzano et al., 2020).

Microbial dysbiosis and associated metabolic alterations are increasingly linked to the initiation and progression of malignancies. Dysbiosis, arising from factors such as diet, antibiotics, and chronic inflammation (Turnbaugh et al., 2009; David et al., 2014), is increasingly understood not only as a shift in microbial composition but also as a functional disruption of microbial metabolic activity. Rather than solely reflecting reduced microbial richness, dysbiosis involves alterations in key metabolic pathways, loss of beneficial functional outputs, and the emergence of metabolite profiles that promote disease progression (Thaiss et al., 2016; Byndloss et al., 2017). These changes contribute to disease states, including cancer.

In colorectal cancer (CRC), dysbiosis is associated with the enrichment of pro-inflammatory and genotoxic microbes such as *Fusobacterium nucleatum*, *Escherichia coli* (pks^+^), and enterotoxigenic *Bacteroides fragilis*, alongside depletion of SCFA-producing taxa such as *Faecalibacterium prausnitzii* and *Bifidobacterium* species (Mima et al., 2016; Pleguezuelos-Manzano et al., 2020; Okumura et al., 2021). These shifts are accompanied by metabolite imbalances, including reduced butyrate and increased secondary bile acids, polyamines, and reactive species, which are associated with DNA damage, activation of Wnt/β-catenin signaling, chronic inflammation, and immune evasion (Yoshimoto et al., 2013; Pleguezuelos-Manzano et al., 2020).

Beyond CRC, dysbiosis-associated metabolic alterations are observed across multiple cancers. In breast cancer, altered bile acid and SCFA metabolism influence immune modulation and estrogen signaling (Banerjee et al., 2018). In hepatocellular carcinoma, increased lipopolysaccharides (LPS), DCA, ammonia, and bile acids contribute to hepatic inflammation, steatosis, and tumor progression (Yoshimoto et al., 2013). Pancreatic cancer studies have similarly demonstrated distinct fecal and plasma metabolite signatures involving altered tryptophan, polyamine, and bile acid pathways (Riquelme et al., 2019). These findings support the role of microbial metabolites as functional intermediaries linking dysbiosis to cancer biology.

Mechanistic studies support that microbial metabolites influence tumorigenesis through diverse pathways. Tumor-suppressive SCFA butyrate promotes epithelial differentiation and apoptosis via histone deacetylase inhibition (HDACs), while indole derivatives regulate aryl hydrocarbon receptor (AhR) signaling and immune responses (Furusawa et al., 2013; Krishnan et al., 2018). Conversely, DCA, polyamines, and microbial genotoxins, such as colibactin, induce oxidative stress, DNA damage, pro-inflammatory NF-κB signaling, and promote cell proliferation and metastasis, as well as DNA adduct formation and chromosomal instability, thereby promoting tumor initiation (Yoshimoto et al., 2013; Pleguezuelos-Manzano et al., 2020). These mechanistic pathways highlight the multifaceted influence of microbial metabolites on tumorigenesis.

Advances in metabolomics and multi-omics technologies have enabled high-resolution profiling of microbiome-derived metabolites across multiple biological matrices (Wittmann et al., 2014; Mayerle et al., 2018). These metabolites are increasingly recognized as non-invasive biomarkers detectable in stool, blood, and urine, with potential applications in cancer detection and stratification (Yoshimoto et al., 2013; Jové et al., 2017; Riquelme et al., 2019).

Despite these advances, significant challenges remain in translating microbiome–metabolite research into clinical applications. Much of the current evidence is cross-sectional and association-based, limiting causal inference, while variability in microbiome composition across populations, diet, and analytical methods complicates biomarker standardization. Addressing these challenges will require longitudinal studies, standardized workflows, and integrative computational frameworks linking microbial metabolism to host molecular pathways (Franzosa et al., 2019; Wirbel et al., 2019; Jobard et al., 2021).

It is important to emphasize that, despite extensive evidence linking microbiome-derived metabolites with cancer, much of the current literature remains predominantly correlative. Establishing causal relationships will require rigorously designed longitudinal studies, mechanistic validation, and controlled experimental investigations in both *in vitro* and *in vivo* systems (Byndloss et al., 2017; Franzosa et al., 2019). Importantly, tumorigenesis in the context of microbiome-derived metabolites is not solely driven by microbial activity but is critically shaped by host factors, including immune responses, genetic background, and systemic metabolic state. These host determinants modulate the impact of microbial metabolites on tumor initiation and progression (Byndloss et al., 2017; Riquelme et al., 2019).

Unlike existing reviews that primarily summarize individual microbiome-cancer associations, this review provides an integrative synthesis linking microbiome-derived metabolite classes with core tumorigenic signaling pathways. It further emphasizes context-dependent effects across different cancer types and incorporates host-microbiome interactions and multi-omics integration to present a systems-level understanding of metabolite-driven tumorigenesis.

**FIGURE 1:**
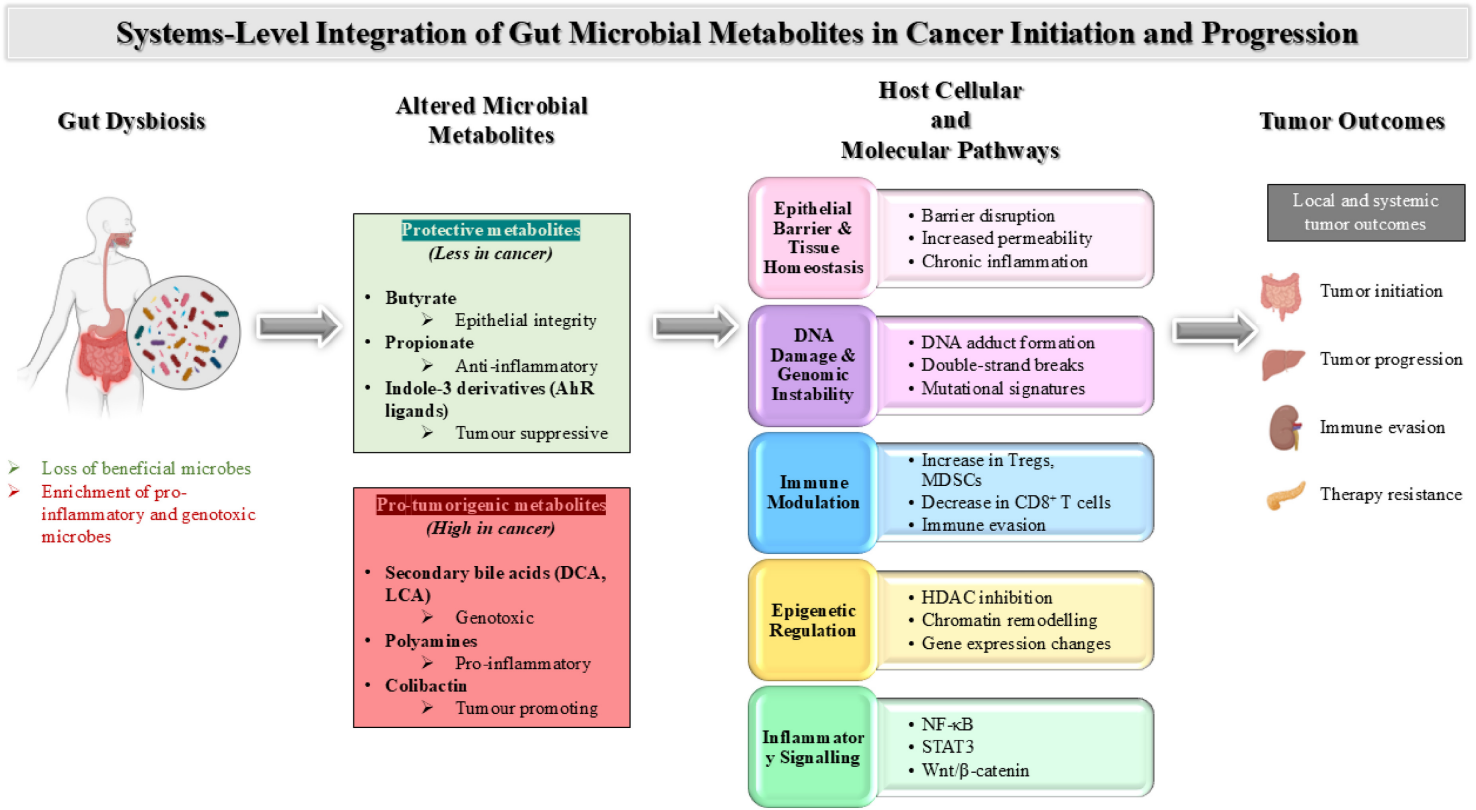
Dysbiosis-driven metabolic alterations in cancer development: Gut microbial dysbiosis alters the composition and functional output of the microbiome, leading to changes in metabolite production. These alterations affect epithelial barrier integrity, immune regulation, inflammatory signaling, and genomic stability, thereby contributing to tumor initiation, progression, immune evasion, and therapeutic resistance.

This review synthesizes current knowledge on microbiome-derived metabolites in cancer biology, examining how dysbiosis-associated metabolic alterations influence tumor initiation, progression, and regression, outlines analytical strategies for characterizing microbial metabolite profiles, and highlights their potential as non-invasive biomarkers and therapeutic targets. Figure 1 summarizes the integrated relationship between gut microbial dysbiosis, altered microbial metabolite production, host cellular signaling pathways, and tumor-associated outcomes involved in cancer biology. Understanding the interplay between microbial metabolism and tumor biology will be critical for advancing microbiome-informed precision oncology.

## 2 Microbiome dysbiosis in cancer

The composition and functional output of the gut microbiome differ markedly between healthy individuals and cancer patients (Yiminniyaze et al., 2025). These alterations are not restricted to gastrointestinal malignancies but are also observed in extra-intestinal cancers, underscoring the systemic influence of gut microbes and their metabolites on host physiology (Riquelme et al., 2019).

Cancer-associated dysbiosis typically involves the depletion of beneficial commensal organisms, enrichment of indigenous or pathogenic taxa, and disruption of microbial metabolic homeostasis (Mima et al., 2016). These changes affect multiple aspects of host physiology, including inflammatory, epithelial barrier function, immune regulation, and microbial metabolite production (Furusawa et al., 2013; Krishnan et al., 2018). Collectively, these alterations contribute to the remodeling of both local and systemic tumor microenvironments (Nejman et al., 2020).

Understanding these dysbiosis shifts is crucial, as they represent the initial functional layer of the microbiome-cancer axis and provide a foundation for the metabolic and mechanistic pathways discussed in the subsequent sections.

### 2.1 Gut microbial dysbiosis in cancer: patterns and biological implications

Cancer-associated dysbiosis is characterized by consistent restructuring of microbial communities, involving reduced diversity, altered microbial abundance, and compromised functional stability (Yiminniyaze et al., 2025). A decline in microbial richness and evenness is frequently observed, reflecting ecosystem instability and the breakdown of the protective microbial networks. The resulting reduction of diversity impairs immunological homeostasis and metabolic balance by weakening colonization resistance against pathogenic and pro-inflammatory species (Spragge et al., 2023).

Notably, dysbiosis does not remain homogenous across all cancer types but indicates both shared and disease-specific characteristics. Due to direct interactions between tumor tissue and gut bacteria, intestinal malignancies, including colorectal and gastric tumors, frequently exhibit localized changes in the microbiome. Conversely, systemic malignancies, such as those of the breast, prostate, and pancreas, typically show dysbiosis patterns that are more influenced by immunological, endocrine, and systemic metabolic disorders than by direct microbial colonization of the tumor site (Golombos et al., 2018; Pushalkar et al., 2018).

Despite these differences, a common feature across cancer types is the emergence of a pro-inflammatory microbial ecosystem that supports tumor growth, facilitates immune evasion, and contributes to metabolic reprograming within the host (Yu et al., 2017).

### 2.2 Qualitative and quantitative shifts in microbial communities

Cancer-associated dysbiosis is defined by both qualitative and quantitative alterations in microbial populations, which disrupt the ecological equilibrium of the gut microbiome and influence host-microbe interactions at several biological levels (Yiminniyaze et al., 2025).

Qualitative alterations include the loss of major beneficial microbes and the appearance of rare or low-abundance microbial species. These changes could result in the loss of protective metabolic mechanisms that are essential for host homeostasis or the introduction of novel microbial functions with pathogenic potential (Zeller et al., 2014).

Quantitative changes refer to alterations in the relative abundance of specific microbial groups, leading to the overgrowth of potentially hazardous taxa or the depletion of beneficial communities. Microbial metabolite synthesis, immunological activation patterns, and epithelial signaling pathways are all significantly affected by these alterations (Krishnan et al., 2018).

Collectively, these qualitative and quantitative modifications support a tumor-promoting microbial environment, thereby influencing cancer progression and therapeutic responsiveness.

#### 2.2.1 Selective enrichment of pro-tumorigenic microbiota in cancer

Numerous microbial taxa have been consistently identified as enriched in individuals with cancer and are increasingly recognized for their potential pro-tumorigenic roles in cancer development (Zeller et al., 2014; Mima et al., 2016). These microbes contribute to carcinogenesis through various mechanisms, including chronic inflammation, production of genotoxic compounds, immune response modulation, and disruption of host metabolic pathways (Yu et al., 2017; Pleguezuelos-Manzano et al., 2020).

In gastrointestinal cancers, increased abundance of species from genera such as *Fusobacterium, Bacteroides, Escherichia*, and *Enterococcus* has been frequently observed (Mima et al., 2016). These organisms interact with epithelial cells, activate pro-inflammatory signaling cascades, and promote DNA damage and immune evasion (Pleguezuelos-Manzano et al., 2020). Certain strains produce toxins, lipopolysaccharides, and genotoxic or pro-inflammatory metabolites that directly or indirectly facilitate tumor growth and progression.

In extra-intestinal cancers, such as pancreatic, liver, and breast cancers, dysbiosis is often characterized by shifts toward gram-negative and endotoxin-producing bacteria (Pushalkar et al., 2018). These microbial changes contribute to systemic low-grade inflammation, increased intestinal permeability, and altered immune signaling, thereby influencing distant organ microenvironments through circulating microbial metabolites and inflammatory mediators (Krishnan et al., 2018). This underlines how gut microbial imbalance can exert tumor-promoting effects beyond the local intestinal environment (Riquelme et al., 2019).

#### 2.2.2 Depletion of commensal taxa with tumor-suppressive potential

In parallel with the proliferation of harmful microorganisms, cancer-associated dysbiosis is characterized by the depletion of beneficial commensal bacteria that play protective roles in maintaining homeostasis (Yiminniyaze et al., 2025). These commensals are integral to preserving epithelial barrier integrity, regulating immune responses, and producing anti-tumorigenic metabolites, particularly short-chain fatty acids (SCFAs) (Furusawa et al., 2013; Krishnan et al., 2018).

A consistent observation across various cancer types is the reduction in the abundance of SCFA-producing bacteria (Shrode et al., 2023). The loss of these organisms compromises mucosal defense, weakens immune surveillance, and weakens the availability of metabolites that typically support apoptosis and inhibit abnormal cell proliferation (Donohoe et al., 2012). This alteration creates an imbalance between pro-inflammatory and anti-inflammatory signals, developing an environment conducive to tumor development (Byndloss et al., 2017).

In addition to SCFA producers, other commensal microbes that modulate the immune system and support epithelial repair are frequently depleted during cancer progression. Their reduction further destabilizes microbial networks and reduces microbial resilience, increasing susceptibility to indigenous and pro-tumorigenic colonization of the gut (Endt et al., 2010). Overall, the simultaneous enrichment of pathogenic taxa and depletion of protective commensals represents a central hallmark of cancer-associated dysbiosis, supporting a microbial environment conducive to tumor initiation, progression, regression and immune evasion (Yiminniyaze et al., 2025).

## 3 Microbial metabolite classes

A key functional aspect of the gut microbiome is microbial metabolism, which acts as a biochemical bridge linking human physiology and microbial communities (Furusawa et al., 2013). Gut microbes metabolize dietary components, host-derived molecules, and xenobiotics into a diverse range of bioactive compounds that influence cellular signaling, immune responses, and tissue homeostasis (Byndloss et al., 2017; Krishnan et al., 2018). In the context of cancer, these metabolites function as critical mediators linking microbial dysbiosis with tumor initiation, progression, or, in certain instances, suppression (Yu et al., 2017; Pleguezuelos-Manzano et al., 2020). Depending on their chemical nature, concentration, and local tissue context, microbe-derived metabolites can either enhance tumor-promoting pathways or support tumor-suppressive mechanisms (Yoshimoto et al., 2013; Krishnan et al., 2018). Understanding the gut microbiome’s role in cancer biology requires an understanding of its functional classes, mechanistic roles, and the specific microorganisms that produce them.

Importantly, dysbiosis should be interpreted as a functional imbalance in microbial metabolic activity rather than solely a reduction in microbial diversity, emphasizing pathway-level disruptions and altered metabolite outputs (Thaiss et al., 2016; Byndloss et al., 2017). Diverse functional classes can be used to classify gut microbial metabolites, many of which are closely related to the microbe metabolite cancer interactions described in Table 1.

**TABLE 1:** Disease correlations, mechanistic functions, and abundance patterns across major malignancies are highlighted by gut microbial species and their main metabolites linked to cancer pathogenesis.

Microbial taxa	Key metabolites	Cancer type	Nature and abundance trend	Mechanistic role
*Fusobacterium nucleatum* subsp. Nucleatum	Polyamines, secondary bile acids	Colorectal cancer	Pro-tumorigenic; typically enriched in CRC patients	Promotes inflammation and immune evasion (Mima et al., 2016; Aymeric et al., 2018; Zhang et al., 2023)
*Escherichia coli* NC101	Colibactin (genotoxin)	Colorectal cancer	Pro-tumorigenic; enriched in CRC patients	Induces DNA damage and mutagenesis (Nougayrède et al., 2006; Bossuet-Greif et al., 2018; Pleguezuelos-Manzano et al., 2020)
*Bacteroides fragilis* (ETBF)	BFT toxin, bile acid metabolites	Colorectal cancer	Pro-tumorigenic; increased abundance in CRC and high-risk lesions	Triggers chronic inflammation and Wnt/β-catenin signaling (Lee et al., 2022; Hwang et al., 2025)
*Clostridium scindens*	Secondary bile acids (e.g., deoxycholic acid)	Liver cancer, CRC-liver axis	Pro-tumorigenic; expanded in dysbiotic states with altered bile acid pools	Induces hepatic inflammation and tumor-promoting microenvironment (Devkota et al., 2012; Yoshimoto et al., 2013)
*Enterococcus faecalis*	Reactive oxygen species, extracellular superoxide	Colorectal cancer	Pro-tumorigenic; increased in CRC and high-risk inflammatory states	Induces oxidative DNA damage and chromosomal instability (Attene-Ramos et al., 2010)
*Peptostreptococcus anaerobius*	Polyamines, pro-inflammatory metabolites	Colorectal cancer	Pro-tumorigenic; enriched in CRC patients	Induces inflammation, activates TLR2/4 signaling, and ROS metabolism (Tsoi et al., 2017; Aymeric et al., 2018; Zhang et al., 2018)
*Fusobacterium nucleatum* subsp. polymorphum	SCFAs, pro-inflammatory metabolites	Breast cancer	Pro-tumorigenic; detected at higher levels in tumor tissue compared with normal	Promotes immune suppression and inflammation (Hieken et al., 2016)
*Prevotella copri* DSM 18205	Branched-chain amino acid metabolites	Pancreatic cancer	Pro-tumorigenic; increased in metabolic dysregulation	Induces inflammation, alters systemic metabolic and immune profiles (Song et al., 2022, 2025)
*Bilophila wadsworthia*	Hydrogen sulfide (H_2_S), bile acid derivatives	Colorectal and liver cancers	Pro-tumorigenic; often enriched under high-fat, bile-rich diets	Enhances epithelial damage and mutagenesis (Devkota et al., 2012; Yoshimoto et al., 2013)
*Streptococcus gallolyticus* subsp. gallolyticus	Secondary bile acids, pro-inflammatory metabolites	Colorectal cancer	Pro-tumorigenic; enriched in CRC and associated with colonic neoplasia	Stimulates inflammatory signaling and epithelial proliferation (Aymeric et al., 2018; Zhang et al., 2018)
*Desulfovibrio piger*	Hydrogen sulfide (H_2_S)	Colorectal cancer	Pro-tumorigenic; increased in dysbiotic, sulfate-reducing microbiomes	Induces epithelial DNA damage and mucosal injury (Attene-Ramos et al., 2010)
*Porphyromonas gingivalis*	SCFAs, gingipains, pro-inflammatory metabolites	Pancreatic and oral cancers	Pro-tumorigenic; over-represented in pancreatic and oral cancer cohorts	Induces chronic inflammation, immune evasion, and proteolytic tissue damage (Mima et al., 2016; Pushalkar et al., 2018; Wang et al., 2019)
*Cutibacterium acnes* (formerly *Propionibacterium acnes*)	Porphyrins, SCFAs	Prostate cancer	Pro-tumorigenic; increased in prostate tumors	Triggers local inflammation and oxidative stress (Olsson et al., 2012; Davidsson et al., 2021)
*Roseburia intestinalis*	Butyrate (SCFA)	Colorectal cancer	Tumor-suppressive; frequently depleted in CRC patients	Promotes epithelial health and induces tumor cell apoptosis (Feng et al., 2018; Furusawa et al., 2013)
*Faecalibacterium prausnitzii* A2-165	Butyrate, anti-inflammatory peptides	Colorectal cancer	Tumor-suppressive; reduced in CRC conditions	Exhibits strong anti-inflammatory effects and suppresses NF-κB-mediated tumor-promoting pathways (Feng et al., 2018; Louis et al., 2010)
*Bifidobacterium longum* subsp. longum	Acetate, folate derivatives	Colorectal, gastric cancers	Tumor-suppressive; typically reduced in healthy microbiomes of cancer patients	Strengthens gut barrier and modulates immune responses (Sivan et al., 2015)
*Lactobacillus reuteri*	Indole-3-aldehyde, SCFAs	Colorectal, breast cancer	Tumor-suppressive; often reduced in cancer compared with healthy controls	Produces anti-inflammatory and barrier-protective metabolites (Zelante et al., 2013; Montgomery et al., 2022; Xu et al., 2023)
*Akkermansia muciniphila* ATCC BAA-835	Acetate, propionate, mucin-derived metabolites	Lung, renal cancers (immune-therapy linked)	Tumor-suppressive; enrichment correlates with better immune-therapy response	Enhances anti-tumor immune responses and improves checkpoint inhibitor efficacy (Derosa et al., 2022)

Acetate, propionate, and butyrate are examples of short-chain fatty acids (SCFAs), which are among the microbial metabolites that have been studied the most. These substances are mostly produced by commensal bacteria via the fermentation of food fibers (Louis et al., 2010). Taxa that produce SCFAs, such as *Roseburia intestinalis* and *Faecalibacterium prausnitzii*, are often depleted in colorectal cancer cases (Feng et al., 2018). This depletion is associated with an accompanying reduction in butyrate levels, which adversely affect epithelial barrier integrity, reduce anti-inflammatory signaling, and impair apoptosis in tumor cells (Furusawa et al., 2013). Butyrate is widely recognized for its role in maintaining epithelial homeostasis and modulating tumor cell behavior through histone deacetylase (HDAC) inhibition. Importantly, the effects of short-chain fatty acids, particularly butyrate, are highly context-dependent. While physiological concentrations promote epithelial homeostasis and anti-inflammatory responses, elevated levels or altered metabolic states in tumor cells result in distinct effects on cell proliferation, differentiation, and apoptosis. This phenomenon is often referred to as the “butyrate paradox,” which highlights how cellular metabolic context influences its functional outcome (Donohoe et al., 2012; Furusawa et al., 2013). Consequently, the loss of these SCFA-producing bacteria contributes to the transition from a protective to tumor-permissive metabolic ecosystem.

Conversely, secondary bile acids are a class of metabolites frequently associated with pro-tumorigenic properties. Bacterial species, such as *Clostridium scindens* and *Bilophila wadsworthia*, are implicated in the conversion of primary bile acids into secondary bile acids, including deoxycholic acid (Devkota et al., 2012; Yoshimoto et al., 2013). These metabolites have the potential to induce oxidative stress, DNA damage, and chronic inflammation, particularly in colorectal and liver cancer. In dysbiosis, the proliferation of these bile acid-modifying taxa alters bile acid pools. It contributes to a hepatic and intestinal microenvironment conducive to tumor development, as evidenced by their enrichment in cancer biology (Devkota et al., 2012). The biological effects of secondary bile acids are also context-dependent and influenced by their concentration, duration of exposure, and tissue-specific microenvironment. While chronic exposure to elevated levels promotes inflammation and DNA damage, physiological levels may participate in normal metabolic signaling pathways (Yoshimoto et al., 2013).

Polyamines are a crucial class of metabolites linked to microbial activity and are implicated in tumor biology (Zhang et al., 2023). Elevated polyamine levels have been observed in association with pro-tumorigenic taxa, including *Fusobacterium nucleatum subsp. nucleatum*, *Peptostreptococcus anaerobius*, and *Streptococcus gallolyticus* (Aymeric et al., 2018; Zhang et al., 2023). These polyamines facilitate rapid cellular proliferation, angiogenesis, and immune suppression, thereby promoting tumor growth and progression. In the context of colorectal cancer, the enrichment of these taxa is associated with increased polyamine production and correlates with a more aggressive and inflammatory tumor microenvironment (Tsoi et al., 2017; Zhang et al., 2018).

Metabolites generated from tryptophan, like indole and its derivatives, are an important signaling center in gut-host communication (Krishnan et al., 2018). Species such as *Lactobacillus reuteri* can produce indole-3-aldehyde and other indole-based molecules that help regulate epithelial homeostasis and immune balance (Montgomery et al., 2022). These metabolites typically exert tumor-suppressive and barrier-protective effects, and the reduction of *Lactobacillus* species in colorectal and breast cancers aligns with the loss of this regulatory function in these cancers (Zelante et al., 2013; Xu et al., 2023). Conversely, in some microbiological situations, dysregulated tryptophan metabolism is associated to prolonged inflammation and immunological tolerance, which indirectly promotes tumor progression (Krishnan et al., 2018).

In addition to these classical metabolite classes, certain microorganisms produce endotoxins, genotoxins, and other carcinogenic compounds. For instance, *Escherichia coli* NC101 and *Escherichia coli* strains harboring the pks^+^ island synthesize colibactin, a genotoxin that induces DNA double-strand breaks and distinctive mutational signatures in colonic epithelial cells (Bossuet-Greif et al., 2018; Pleguezuelos-Manzano et al., 2020). *Enterotoxigenic Bacteroides fragilis* (ETBF) produces BFT and bile acid metabolites that activate inflammation and Wnt/β-catenin signaling, thereby facilitating tumor initiation (Lee et al., 2022). Sulfate-reducing bacteria, such as *Desulfovibrio piger*, produce hydrogen sulfide, a genotoxic metabolite that damages epithelial DNA and compromises mucosal integrity (Attene-Ramos et al., 2010).

Several commensal taxa, conversely, have shown metabolic actions that prevent tumor growth. Derivatives of acetate and folate produced by *Bifidobacterium longum* subsp. longum improves barrier function and stimulates immune responses against tumors (Sivan et al., 2015). By stimulating tumor immunity and host metabolic reprograming, *Akkermansia muciniphila* produces metabolites such as acetate, propionate, and mucin and has been linked to better responses to immunotherapy in lung cancer (Derosa et al., 2022). Similarly, *Lactobacillus reuteri* and other lactic acid bacteria produce anti-inflammatory metabolites and strengthen epithelial defenses (Brandi et al., 2020).

*Fusobacterium nucleatum*, *Bilophila wadsworthia*, *Desulfovibrio piger*, *Porphyromonas gingivalis*, and *Cutibacterium acnes* are a few examples of microbes that are clearly pro-tumorigenic, while *Faecalibacterium prausnitzii*, *Roseburia intestinalis*, *Bifidobacterium longum*, and *Akkermansia muciniphila* are a few examples of microbes that are noticeably tumor-suppressive (Mima et al., 2016). This equilibrium between harmful and protective taxa, along with their metabolite profiles, is a defining characteristic of cancer-associated dysbiosis (Riquelme et al., 2019; Yiminniyaze et al., 2025). Collectively, these metabolite classes exert their biological effects through a shared set of signaling pathways, including inflammatory cascades, epigenetic regulation, and immune modulation. Understanding how these metabolites converge on common tumorigenic mechanisms is essential for integrating microbiome-derived metabolic activity into a unified model of cancer progression (Donohoe et al., 2012; Yoshimoto et al., 2013). Thus, the functional effects of microbial metabolites are not strictly linear but are shaped by multiple factors, including concentration gradients, local tissue microenvironment, host metabolic status, and disease stage. This complexity underscores the need for context-specific interpretation of metabolite activity in cancer (Donohoe et al., 2012; Riquelme et al., 2019). While individual metabolite classes have been extensively studied, integrating their collective effects provides a more comprehensive understanding of how microbiome-derived metabolites function as a coordinated network influencing tumorigenesis. These observations reinforce that cancer-associated dysbiosis is best defined by functional and metabolic disruption rather than broad ecological indices such as microbial richness alone (Thaiss et al., 2016; Byndloss et al., 2017).

## 4 Mechanistic pathways in tumorigenesis

**FIGURE 2:**
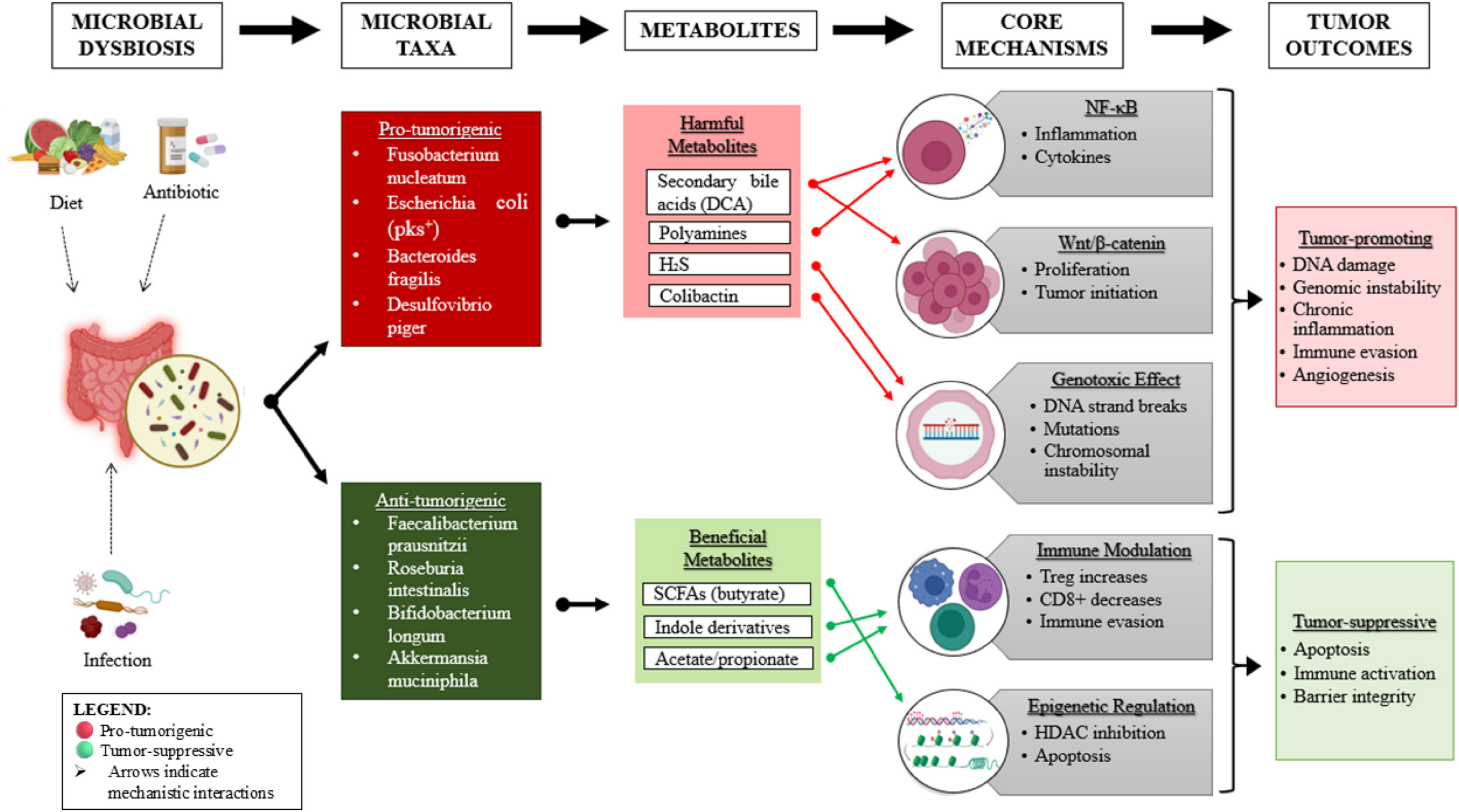
Mechanistic pathways of microbiome-derived metabolites in tumorigenesis: Microbial dysbiosis leads to altered production of metabolites that regulate key host signaling pathways. Pro-tumorigenic metabolites, including secondary bile acids, polyamines, hydrogen sulfide, and genotoxins, activate NF-κB-mediated inflammation, Wnt/β-catenin signaling, and immune-suppressive pathways, promoting tumor progression and genomic instability. In contrast, beneficial metabolites such as short-chain fatty acids and indole derivatives regulate epigenetic modifications, enhance anti-tumor immune responses, and maintain epithelial integrity. Certain metabolites, including colibactin and hydrogen sulfide, directly induce DNA damage and chromosomal instability through genotoxic effects independent of canonical signaling pathways.

Microbiome-derived metabolites do not act in isolation but converge on a limited set of core tumorigenic signaling pathways. Major metabolite classes, including short-chain fatty acids, secondary bile acids, polyamines, and indole derivatives, collectively influence key regulatory axes such as NF-κB-mediated inflammation, Wnt/β-catenin signaling, epigenetic modulation through histone deacetylase inhibition, and immune checkpoint regulation (Yoshimoto et al., 2013; Pleguezuelos-Manzano et al., 2020). This framework moves beyond individual metabolite descriptions by integrating diverse metabolite classes into a unified model of tumorigenic signaling. This integrative pathway provides a mechanistic basis linking microbial metabolic activity with tumor initiation, progression, and immune evasion across different cancer types (Yoshimoto et al., 2013; Pleguezuelos-Manzano et al., 2020). A schematic overview illustrating the interplay between microbiome-derived metabolites and host signaling mechanisms encompassing NF-κB activation, Wnt/β-catenin signaling, immune modulation, epigenetic regulation, and direct genotoxic effects is described in Figure 2.

### 4.1 Axes of influence: microbes, metabolites, and malignancy

In colorectal cancer, the presence of *Fusobacterium nucleatum subsp. nucleatum* and *Peptostreptococcus anaerobius* are notably increased, correlating with the elevated production of polyamines and pro-inflammatory metabolites (Mima et al., 2016; Tsoi et al., 2017; Zhang et al., 2023). These microbial taxa facilitate tumor progression by adhering to epithelial cells, activating inflammatory pathways and Toll-like receptor signaling, and promoting immune evasion (Mima et al., 2016; Chen et al., 2020). Additionally, *Escherichia coli* NC101 and pks^+^ strains contribute to genotoxic stress by producing colibactin, which directly induces DNA damage (Pleguezuelos-Manzano et al., 2020). Simultaneously, sulfate-reducing bacteria, such as *Desulfovibrio piger*, produce hydrogen sulfide, aggravating genotoxic and mucosal injuries (Kushkevych et al., 2019). Furthermore, bile acid-modifying microbes, including *Clostridium scindens* and *Bilophila wadsworthia*, alter the bile acid pool toward profiles that promote tumor development (Devkota et al., 2012; Yoshimoto et al., 2013).

In breast cancer, *Fusobacterium nucleatum* subsp. *Polymorphum* has been observed in greater abundance in tumor tissues than in adjacent normal tissues (Hieken et al., 2016). Its metabolic byproducts and pro-inflammatory potential are believed to contribute to local immune suppression and tumor progression in the tumor micro-environment (TME) (Gur et al., 2015). In contrast, *Lactobacillus reuteri* may mitigate these effects by producing short-chain fatty acids (SCFAs) and indoles (Krishnan et al., 2018). Although its abundance is frequently decreased in malignant conditions, suggesting a lack of protective metabolic activity.

*Porphyromonas gingivalis* has been identified as a significant taxon in both pancreatic and oral cancers (Pushalkar et al., 2018). It produces gingipains, a class of cysteine proteases, short-chain fatty acids (SCFAs), and other proinflammatory metabolites that have been linked to tissue damage, immune evasion, and chronic inflammation (Wang et al., 2019). Its presence in the oral cavity and association with systemic cancers highlight the critical role of extra-intestinal microbial reservoirs in influencing cancer risk (Pushalkar et al., 2018).

*Cutibacterium acnes*, formerly known as *Propionibacterium acnes*, has been linked to oxidative stress and local inflammation in the context of prostate cancer (Davidsson et al., 2021). This is achieved by producing porphyrins and short-chain fatty acids (SCFAs), which contribute to the establishment of a tumor-permissive microenvironment in the TME (Olsson et al., 2012).

However, tumor-suppressive taxa like *Faecalibacterium prausnitzii*, *Bifidobacterium longum*, and *Roseburia intestinalis* are frequently reduced in malignancies when beneficial microbial functions are impaired (Yusuf et al., 2019; Dikeocha et al., 2022). This depletion has been reported to reduce levels of butyrate, acetate, and anti-inflammatory peptides, which, in turn, weaken epithelial barrier function, diminish apoptotic and anti-proliferative signaling in tumor cells, and reduce immune tolerance to malignant cells (Donohoe et al., 2012; Furusawa et al., 2013; Krishnan et al., 2018). *Akkermansia muciniphila* illustrates a metabolically beneficial species whose enrichment in certain clinical contexts is associated with enhanced immunotherapy responses, particularly in lung and renal cancer (Salgia et al., 2020).

Collectively, these microbial taxa exert their tumor-promoting or tumor-suppressive effects through common mechanistic pathways, including NF-κB-mediated inflammation, Wnt/β-catenin signaling, epigenetic modulation, and immune regulation. This highlights how diverse microbiome-derived metabolites converge on shared molecular processes driving tumorigenesis (Pleguezuelos-Manzano et al., 2020).

A comprehensive framework that links particular taxa with their major metabolites, related cancer types, and the specificity of their effects, whether pro-tumorigenic or tumor-suppressive, is demonstrated in Table 1. The underlying mechanistic themes of these patterns are explored in detail in the subsequent section.

### 4.2 Key mechanistic pathways in microbial metabolite-mediated tumor regulation

**FIGURE 3:**
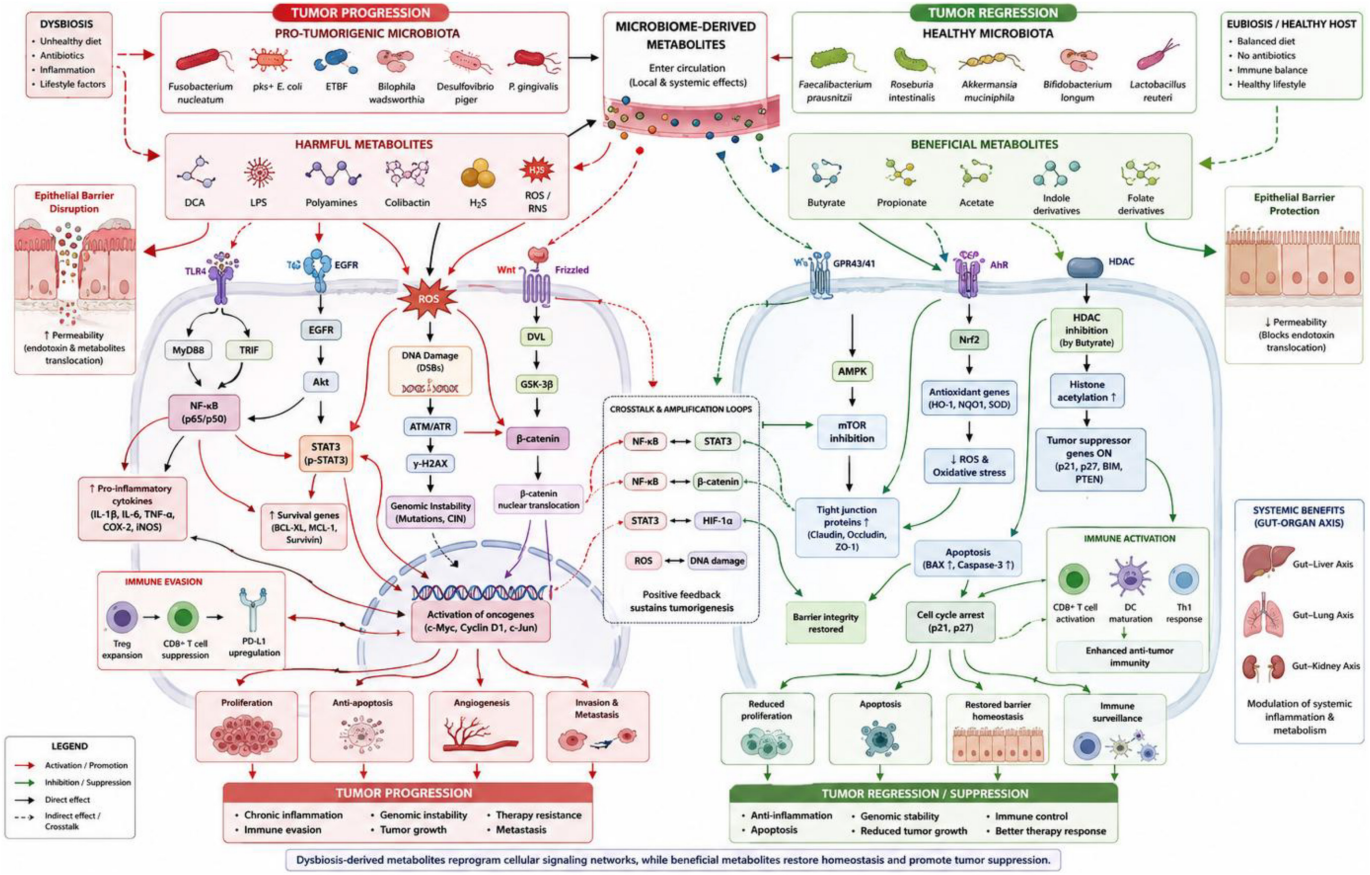
Integrated hybrid signaling network illustrating microbiome-derived metabolite-mediated tumor progression and tumor suppression pathways: Cancer-associated dysbiosis promotes the enrichment of pro-tumorigenic microbial taxa, leading to the production of harmful metabolites including secondary bile acids, lipopolysaccharides, polyamines, hydrogen sulfide, and colibactin. These metabolites activate interconnected cellular signaling pathways involving (TLR)/NF-κB signaling, EGFR/STAT3 activation, Wnt/β-catenin signaling, oxidative stress, DNA damage, genomic instability, immune evasion, and inflammatory cytokine production, collectively contributing to tumor initiation, proliferation, angiogenesis, metastasis, and therapy resistance. In contrast, beneficial commensal microbiota produce tumor-suppressive metabolites such as short-chain fatty acids, indole derivatives, acetate, propionate, and folate derivatives, which promote epithelial barrier integrity, HDAC inhibition, AhR signaling, antioxidant responses, apoptosis, cell-cycle arrest, and anti-tumor immune activation. The figure highlights the integrated crosstalk between microbial metabolites, host signaling pathways, immune regulation, and tumor microenvironment dynamics that collectively determine cancer progression or regression across multiple organ systems.

Figure 3 illustrates an integrated hybrid mechanistic framework demonstrating how microbiome-derived metabolites regulate tumor progression or tumor suppression through interconnected cellular signaling pathways within the tumor microenvironment. Rather than functioning through isolated mechanisms, microbial metabolites converge on inflammatory, oncogenic, immune, epigenetic, and metabolic signaling networks that collectively shape tumor behavior.

In the tumor-promoting axis, cancer-associated dysbiosis enriches pro-tumorigenic microbial taxa including *Fusobacterium nucleatum*, pks^+^ *Escherichia coli*, *Enterotoxigenic Bacteroides fragilis* (ETBF), *Bilophila wadsworthia*, and *Desulfovibrio piger*. These microorganisms produce harmful metabolites such as secondary bile acids, lipopolysaccharides (LPS), polyamines, hydrogen sulfide (H_2_S), reactive oxygen species (ROS), and colibactin, which disrupt epithelial barrier integrity and activate multiple tumorigenic signaling pathways (Yoshimoto et al., 2013; Pleguezuelos-Manzano et al., 2020). LPS-mediated activation of Toll-like receptor 4 (TLR4) stimulates Nuclear Factor-kappa B (NF-κB) signaling and induces pro-inflammatory cytokines including Interleukin-6 (IL-6) and Tumor Necrosis Factor-α (TNF-α), thereby sustaining chronic inflammation (Yu et al., 2017; Krishnan et al., 2018). Simultaneously, genotoxic metabolites such as colibactin and H_2_S induce oxidative stress, DNA damage, and genomic instability (Bossuet-Greif et al., 2018; Pleguezuelos-Manzano et al., 2020). These inflammatory and genotoxic events further activate oncogenic pathways including Wnt/β-catenin and Epidermal Growth Factor Receptor/Signal Transducer and Activator of Transcription 3 (EGFR/STAT3), promoting tumor cell proliferation, angiogenesis, metastasis, and immune evasion through Programed Death-Ligand 1 (PD-L1) upregulation and suppression of cytotoxic CD8^+^ T-cell activity (Yoshimoto et al., 2013; Lee et al., 2022; Zhang et al., 2023).

Conversely, the tumor-suppressive axis is mediated by beneficial commensal microbiota including *Faecalibacterium prausnitzii*, *Roseburia intestinalis*, *Akkermansia muciniphila*, *Bifidobacterium longum*, and *Lactobacillus reuteri*. These microbes generate protective metabolites such as short-chain fatty acids (SCFAs), particularly butyrate, acetate, and propionate, along with indole derivatives and folate-associated metabolites (Furusawa et al., 2013). SCFAs enhance epithelial barrier integrity and regulate cellular metabolism through G-protein coupled receptor signaling and AMP-activated Protein Kinase (AMPK)-mediated pathways. Butyrate additionally functions as a Histone Deacetylase (HDAC) inhibitor, promoting tumor suppressor gene activation, apoptosis, and cell-cycle arrest (Donohoe et al., 2012). Indole derivatives activate Aryl Hydrocarbon Receptor (AhR)-mediated signaling, which supports antioxidant responses, mucosal immune homeostasis, and anti-inflammatory activity. Beneficial microbial metabolites also enhance dendritic cell maturation and CD8^+^ T-cell activation, thereby strengthening anti-tumor immune surveillance.

Collectively, Figure 3 highlights that microbiome-derived metabolites play a central role in regulating cellular proliferation, apoptosis, and metabolic reprograming (Donohoe et al., 2012; Krishnan et al., 2018). These metabolites can either stimulate uncontrolled tumor cell proliferation or cause apoptosis and metabolic reprograming or disruption in malignant cells by interacting with host nutrient-sensing and growth-regulatory mechanisms (Byndloss et al., 2017). These metabolic interactions highlight the significant influence of microbial metabolites as potent modulators of tumor behavior in various cancer types, with multiple metabolite classes acting through overlapping signaling networks rather than isolated mechanisms (Yu et al., 2017; Pleguezuelos-Manzano et al., 2020). Building upon these metabolic alterations, microbial metabolites influence tumorigenesis through several interconnected signaling pathways. Although mechanistic evidence from experimental models is increasing, much of the existing literature remains predominantly observational. As a result, distinguishing causality from association remains a critical and unresolved challenge in microbiome-cancer research (Franzosa et al., 2019; Wirbel et al., 2019).

## 5 Host-microbiome crosstalk in tumorigenesis

Through metabolic, immunological, and cellular signaling pathways, the gut microbiota communicates regularly with host tissues rather than functioning as a separate biological unit. This bidirectional interaction is strongly influenced by host determinants, including immune responses, genetic background, and metabolic state, which collectively shape the functional outcomes of microbiome-derived metabolites (Krishnan et al., 2018). This dynamic interaction, known as host-microbiome crosstalk, plays a crucial role in maintaining physiological homeostasis and is significantly dysregulated during cancer development (Mima et al., 2016).

Significant regulators of this interaction, microbial metabolites have an impact on systemic immune responses, genomic stability, and tumor microenvironment dynamics (Furusawa et al., 2013; Pleguezuelos-Manzano et al., 2020). Through these mechanisms, gut microbial communities can influence both local and distant tumor behavior (Riquelme et al., 2019). Thus, understanding this crosstalk is crucial for linking microbial alterations with functional and biological outcomes in cancer (Pleguezuelos-Manzano et al., 2020).

### 5.1 Microbial metabolite signaling in the tumor microenvironment

The tumor microenvironment is a dynamic and intricate ecosystem that includes stromal cells, immunological infiltrates, vascular networks, extracellular matrix components, and malignant cells. This environment is significantly influenced by microbial metabolites, which alter inflammatory signaling, metabolic condition, immune cell recruitment, and cellular communication (Krishnan et al., 2018; Pleguezuelos-Manzano et al., 2020).

Microbial metabolites directly interact with epithelial and immune cell receptors, initiating downstream signaling pathways that modify cytokine secretion profiles, immune surveillance, and angiogenic responses (Krishnan et al., 2018). Certain metabolites enhance anti-tumor immune activity by facilitating the recruitment and function of cytotoxic immune cells (Sivan et al., 2015; Riquelme et al., 2019). Other metabolites, conversely, encourage the growth of immunosuppressive cell populations, which makes immune evasion easier and increases tumor survival (Yu et al., 2017).

In addition to their role in immune signaling, microbial metabolites significantly influence the metabolic framework of the tumor microenvironment. By modifying nutrient availability, oxygen consumption, and energy metabolism, these metabolites enable metabolic reprograming, a fundamental characteristic of cancer that enables tumor cells to adapt and continue under conditions of hypoxia, oxidative stress, and nutrient scarcity (Donohoe et al., 2012; Byndloss et al., 2017).

### 5.2 Circulatory dynamics of microbial metabolites and their organ-level impacts

Microbial metabolites originating from the gut are not restricted to the intestinal tracts (Byndloss et al., 2017). A significant number of these molecules pass through the intestinal epithelial barrier and enter the circulation, facilitating their distribution to remote organs (Venkatesh et al., 2014). Upon absorption, these metabolites initially enter the circulation system, exerting a direct influence on the liver before being released into the systemic circulation and reaching organs such as the lungs, pancreas, breasts, prostate, and kidneys (Yoshimoto et al., 2013).

Hepatocytes connected directly to systemic circulation are more susceptible to endotoxins and microbial metabolites in liver cancer. This exposure triggers local inflammation, fibrosis, immune dysregulation, and, ultimately, malignant transformation (Yoshimoto et al., 2013). Whereas, in extra-intestinal malignancies, such as breast and prostate cancers, circulating microbial metabolites may influence hormone metabolism, immune responses, and tumor-associated metabolic pathways (Banerjee et al., 2018).

The systemic effects explain how gut microbial activity supports a vast metabolic and immunological network, influencing tumor behavior at distant anatomical sites and exerting its influence outside of the gastrointestinal tract (Riquelme et al., 2019).

### 5.3 Metabolite-induced alterations in genomic stability and cellular homeostasis

An essential consequence of host-microbiome interactions is their capacity to affect genomic stability and cellular destiny of the host. Certain microbial metabolites exhibit genotoxic properties or induce persistent oxidative stress, leading to DNA damage, mutations, chromosomal instability, and the progressive accumulation of oncogenic alterations (Attene-Ramos et al., 2010; Pleguezuelos-Manzano et al., 2020).

Microbial metabolites also influence fundamental cellular pathways that regulate proliferation, apoptosis, and DNA repair (Krishnan et al., 2018). Certain metabolites facilitate unregulated cellular growth by activating growth and survival pathways or compromising checkpoint mechanisms (Yoshimoto et al., 2013). In contrast promote programed cell death and growth arrest by engaging in pro-apoptotic signaling cascades (Krishnan et al., 2018).

Significantly, the influence of these metabolites extends beyond tumor cells, affecting adjacent non-malignant cells by modifying DNA repair mechanisms, stress response pathways, and tissue homeostasis (Pleguezuelos-Manzano et al., 2020). This extensive impact plays a crucial role in establishing a microenvironment that either supports or restricts tumor growth, depending on the main metabolite profile (Riquelme et al., 2019).

## 6 Multi-omics analytical approaches

Understanding the intricate relationship between the microbiome and its metabolic output in cancer requires sophisticated analytical frameworks that capture both microbial composition and functional activity (Franzosa et al., 2019; Jiang et al., 2025). Technological advancements in sequencing and metabolomics have significantly enhanced our capacity to analyze these interactions with greater depth, resolution, and precision. Each analytical method offers a unique layer of biological information, and their integration is crucial for generating comprehensive insights into cancer-associated microbiome alterations and metabolite dynamics (Zeller et al., 2014; Jiang et al., 2025).

### 6.1 Multi-layered microbial profiling

Microbial community profiling is predominantly performed using 16S rRNA gene sequencing, which elucidates the taxonomic composition of bacterial populations (Qin et al., 2010). This method facilitates the identification of community diversity, relative abundance of taxa, and microbial signatures associated with cancer (Kostic et al., 2012). Despite being extensively utilized and reasonably priced, 16S sequencing only offers an inadequate number of functional insights because of its low resolution, which is usually restricted to the genus or species level.

Shotgun metagenomic sequencing addresses these limitations by facilitating strain-level identification and enabling the direct analysis of the genetic potential inherent in microbial communities (Franzosa et al., 2019). This analytical method allows researchers to identify functional genes associated with metabolite production, toxin synthesis, and host interaction mechanisms. Consequently, metagenomics has played an essential role in associating specific microbial species with functional pathways relevant to carcinogenesis (Zeller et al., 2014; Pleguezuelos-Manzano et al., 2020). However, despite these advantages, integrating 16S rRNA sequencing and shotgun metagenomics with metabolomic datasets remains a significant computational challenge. Differences in sequencing depth, taxonomic resolution, data normalization, and feature annotation across platforms introduce substantial variability, limiting cross-study comparability and biological interpretation (Franzosa et al., 2019; Wirbel et al., 2019).

A major limitation in current multi-omics studies is the lack of standardized computational pipelines for high-resolution integration of metagenomic and metabolomic data. Variability in preprocessing workflows, bioinformatic tools, and statistical frameworks often results in inconsistent outputs, thereby affecting reproducibility and the robustness of inferred biological relationships (Franzosa et al., 2019; Wirbel et al., 2019).

Meta-transcriptomics enhances this analysis by capturing actively expressed microbial genes, thereby providing real-time insights into functional activity rather than merely genomic potential (Franzosa et al., 2019). Collectively, these methodologies provide a comprehensive view of microbial structure and function, facilitating the correlation between microbial alterations and cancer-associated metabolic and immune changes.

Furthermore, translating these integrated datasets into mechanistic insights remains challenging, as linking microbial taxa to specific metabolite functions and host responses requires advanced computational modeling and experimental validation. This limitation highlights a critical gap between data generation and biological interpretation in current microbiome research (Franzosa et al., 2019).

In addition to high-throughput sequencing methods, several targeted and microscopy-based techniques have been widely used to validate microbial findings and analyze spatial distribution within cancer-associated microbiomes (Riquelme et al., 2019). Quantitative PCR (qPCR) is often used to measure specific bacterial taxa or functional genes related to metabolites, offering high sensitivity and rapid validation of sequencing results. This method is especially useful for studying known cancer-associated microbes or specific biosynthetic pathways related to microbial metabolite production.

Direct visualization of microorganisms in tissue sections and biofilms is made possible by Fluorescence *In Situ* Hybridization (FISH), which offers spatial context that sequencing techniques are unable to provide (Nejman et al., 2020). This technique has been employed to investigate bacterial colonization in tumor tissues and mucosal layers, particularly in colorectal and gastric cancers (Mima et al., 2016).

Culturomics is a culture-based methodology that complements sequencing by enabling the isolation and physiological characterization of live microorganisms from clinical samples. It provides high-throughput. This approach is particularly advantageous for investigating functional metabolite production and host-microbe interactions under controlled laboratory conditions (Lagier et al., 2015).

### 6.2 Advanced analytical platforms for microbial metabolomic profiling

Metabolomics includes liquid chromatography-mass spectrometry (LC-MS), gas chromatography-mass spectrometry (GC-MS), and nuclear magnetic resonance (NMR) spectroscopy. They are extensively utilized for the direct analysis of microbial metabolite production (Wikoff et al., 2009). These methodologies facilitate the detection and quantification of small molecules in biological samples, such as stool, plasma, urine, and tissue specimens (Marcobal et al., 2013).

Liquid chromatography-mass spectrometry (LC-MS) is particularly advantageous for profiling a diverse range of polar and non-polar metabolites with high sensitivity (Dunn et al., 2011). Gas chromatography-mass spectrometry (GC-MS) is frequently used to analyze volatile compounds and short-chain fatty acids due to its high resolution and reproducibility. Nuclear magnetic resonance (NMR) spectroscopy, although less sensitive than mass spectrometry, provides highly reproducible and non-destructive analyses with minimal sample preparation and strong quantitative reliability (Psychogios et al., 2011).

When these analytical platforms are integrated, they enable comprehensive characterization of metabolite profiles in both malignant and non-cancerous conditions. However, the application of microbiome-derived metabolites as clinical biomarkers is challenged by substantial inter-individual variability driven by differences in diet, microbiome composition, host genetics, and environmental exposures. In addition, many metabolites exhibit diurnal and temporal fluctuations, which significantly affect reproducibility and complicate standardization across studies. These factors limit the reliability of metabolite-based biomarkers for routine clinical diagnostics (Wittmann et al., 2014; Jobard et al., 2021).

Consequently, the establishment of standardized sampling protocols, time-controlled study designs, and robust normalization strategies is essential to improve the clinical applicability of microbiome-derived metabolite biomarkers (Thomas et al., 2019).

In addition to conventional LC-MS and GC-MS platforms, advanced mass spectrometry techniques, such as Matrix-Assisted Laser Desorption/Ionization Mass Spectrometry Imaging (MALDI-MSI), have emerged as potent methodologies for examining the spatial distribution of metabolites within tissues (Eberlin et al., 2014). MALDI-MSI enables the visualization of metabolite localization directly within tumors and adjacent tissues, providing insights into tumor metabolism and the heterogeneity of the microenvironment (Calligaris et al., 2015).

Furthermore, targeted metabolomics platforms are increasingly being employed for the absolute quantification of predefined microbial metabolites, including bile acids, short-chain fatty acids, polyamines, and tryptophan derivatives (Wikoff et al., 2009; Jiang et al., 2025). These platforms are instrumental in validating candidate biomarkers during clinical translation phases; however, their successful implementation requires careful consideration of biological variability, temporal dynamics, and standardization constraints (Thomas et al., 2019; Jobard et al., 2021).

### 6.3 Computational integration of multi-omics data

While individual omics approaches yield valuable data, research on cancer-related microbiomes increasingly depends on integrating multiple data output results (Song et al., 2022, 2025). Multi-omics integration combines microbiome sequencing data with metabolomics, transcriptomics, proteomics, and clinical datasets to provide a systems-level understanding of tumor-microbiome interactions (Jiang et al., 2025).

Advanced computational tools and machine learning techniques are employed to analyze these complex datasets, identify patterns, construct predictive models, and discover metabolite signatures associated with disease states (Zhu et al., 2023; Jiang et al., 2025). These techniques make it easier to distinguish between correlations and causative interactions, classify patient groupings according to metabolic and microbiological profiles, and find possible therapeutic targets or biomarkers (Zhu et al., 2023).

Despite its significant potential, multi-omics integration is constrained by several challenges, including data heterogeneity across platforms, batch effects, limited standardization of analytical pipelines, and difficulties in integrating multi-layered datasets. Furthermore, interpretation of complex multi-omics outputs remains challenging, often limiting biological inference and clinical translation (Franzosa et al., 2019; Thomas et al., 2019). These limitations highlight the need for robust computational frameworks, standardized workflows, and improved methods for cross-platform data integration to enhance the reliability and interpretability of multi-omics analyses in cancer research (Thomas et al., 2019). Improving the reproducibility and translational relevance of microbiome-based cancer research requires addressing these problems (Wirbel et al., 2019). An overview of key sequencing, metabolomic, and integrative analytical platforms used to investigate microbiome-derived metabolites in cancer research is summarized in Table 2.

**TABLE 2:** Overview of sequencing, metabolomic, and integrative analytical platforms used to investigate microbiome-derived metabolite interactions in cancer research and biomarker development.

Technique	Target	Sample	Advantage	Limitation	Key output
16S rRNA gene sequencing	Bacterial taxonomic profiles	Stool/tissue	Cost-effective, high-throughput	Limited functional insights	Characterizing dysbiosis patterns in cancer patients (Qin et al., 2010; Kostic et al., 2012)
Shotgun metagenomics	Whole microbial genomes	Stool/tissue	High resolution, functional prediction	Expensive, computationally heavy	Linking microbial genes with metabolite production and cancer progression (Zeller et al., 2014; Franzosa et al., 2019; Pleguezuelos-Manzano et al., 2020)
Meta-transcriptomics	Microbial RNA	Stool/tissue	Reflects functional activity	RNA instability, high cost	Identifying metabolically active taxa in tumor-adjacent microbiota (Franzosa et al., 2019)
Liquid chromatography-mass spectrometry	Broad range of metabolites	Plasma/serum/stool/urine	High sensitivity, wide metabolite range	Requires complex sample prep	Potential biomarker discovery and metabolic pathway analysis (Wikoff et al., 2009; Dunn et al., 2011)
Gas chromatography-mass spectrometry	Volatile and SCFAs	Stool/serum	High resolution for small metabolites	Limited to volatile compounds	SCFA alterations in CRC and gut dysbiosis (Dunn et al., 2011; Psychogios et al., 2011)
Nuclear magnetic resonance spectroscopy	Global metabolite fingerprint	Serum/urine/tissue	Highly reproducible, non-destructive	Lower sensitivity than MS	Longitudinal metabolomic profiling in cancer (Psychogios et al., 2011)
Quantitative PCR	Specific microbial taxa	Stool/tissue	Highly sensitive, fast validation	Limited to predefined targets	Validation of cancer-associated microbes and metabolite-producing genes (Kostic et al., 2012; Banerjee et al., 2018)
Fluorescence *In Situ* hybridization	Spatial microbial localization	Tissue	Visual and spatial resolution	Low throughput, limited probes	Localization of bacteria in the tumor micro-environment (Mima et al., 2016; Nejman et al., 2020)
Culturomics	Viable microbial isolates	Stool, tissue	Allows functional/metabolic tests	Culture bias, not all microbes grow	Studying metabolite production and host-microbe interaction (Lagier et al., 2015)
Matrix-assisted laser desorption/ionization mass spectrometry imaging	Spatial metabolite distribution	Tumor tissue	Spatial metabolomics, high resolution	Technically demanding, costly	Mapping tumor metabolic heterogeneity (Eberlin et al., 2014; Calligaris et al., 2015)
Targeted metabolomics panels	Specific microbial metabolites	Plasma, urine, stool	High specificity and clinical relevance	Limited to predefined metabolites	Clinical validation of metabolite biomarkers (Wikoff et al., 2009; Jiang et al., 2025; Thomas et al., 2019; Jobard et al., 2021)
Multi-omics integration platforms	Integrated microbiome and metabolomics	Stool/urine/blood	Captures complex interactions	Requires advanced bioinformatics	Systems biology interpretation in cancer-microbiome studies (Jiang et al., 2025; Song et al., 2025)
Artificial intelligence and machine learning	Omics datasets	Combined datasets	High predictive power	Overfitting risk needs large datasets	Cancer classification and biomarker prediction from microbiome metabolome data (Zhu et al., 2023; Jiang et al., 2025)

## 7 Cancer-specific metabolite signatures

Although common mechanistic themes are observed across malignancies, the effects of microbiome-derived metabolites are highly tissue-specific and influenced by differences in local metabolism, immune micro-environment, and organ-specific physiology. These variations result in distinct metabolite-driven pathways across different cancer types (Riquelme et al., 2019; Salgia et al., 2020). While common mechanistic themes are observed across various cancers, each tumor type displays a unique pattern of microbial dysbiosis and metabolite alteration (Riquelme et al., 2019; Nejman et al., 2020). Understanding these cancer-specific signatures is crucial for identifying targeted potential biomarkers and understanding how the same classes of metabolites can have distinct effects in different tissue contexts (Song et al., 2022). The following subsections provide a summary of the key microbiome-metabolite patterns across major cancers, organized from the most commonly diagnosed to those with emerging but less extensive evidence (Song et al., 2022, 2025).

### 7.1 Lung cancer

Lung cancer continues to be the primary cause of cancer-related mortality worldwide, with approximately 2.5 million new cases and 1.8 million deaths reported in 2022. The age-standardized incidence rate (ASR) is 31.5 per 100,000, while the mortality rate is 22.5 per 100,000 (Bray et al., 2024). Traditionally linked to tobacco exposure and environmental pollutants, lung cancer is increasingly understood as a condition influenced by systemic inflammation and immune dysregulation.

Recent studies have identified a functional gut-lung axis, in which metabolites derived from the gut microbiome modulate the pulmonary immune tone and inflammatory responses (Yiminniyaze et al., 2025). Alterations in the microbial metabolism of tryptophan were particularly significant, as shown in Table 3 (Zelante et al., 2013; Krishnan et al., 2018). In lung cancer, reduced circulating levels of indole and its derivatives have been observed, indicating impaired tryptophan metabolism (Yiminniyaze et al., 2025). These metabolites are crucial regulators of mucosal immunity and epithelial integrity through aryl hydrocarbon receptor (AhR) signaling (Zelante et al., 2013; Krishnan et al., 2018). Their depletion may compromise immune surveillance and substitute a pro-inflammatory environment beneficial to tumor initiation and progression (Krishnan et al., 2018; Yiminniyaze et al., 2025).

**TABLE 3:** Cancer-specific microbial metabolite biomarkers across major malignancies, highlighting their direction of alteration, functional role in tumor biology, sample source, and potential clinical relevance.

Cancer type	Metabolite	Level	Role	Sample	Clinical use
Colorectal cancer	Deoxycholic acid (secondary bile acid)	High	Pro-tumor	Feces/serum	Early detection, adenoma-carcinoma progression marker (Yoshimoto et al., 2013; Donohoe et al., 2012; Pleguezuelos-Manzano et al., 2020)
Colorectal cancer	Butyrate	Low	Anti-tumor	Feces	Indicates loss of protective microbes and tumor progression (Donohoe et al., 2012; Yoshimoto et al., 2013; Mima et al., 2016)
Breast cancer	Estrogen-like microbial metabolites	High	Pro-tumor	Serum	Associated with hormone-dependent tumor progression (Banerjee et al., 2018; Hieken et al., 2016; Krishnan et al., 2018)
Lung cancer	Indole derivatives	Low	Anti-tumor	Plasma	Linked to impaired immune regulation and tumor progression (Zelante et al., 2013; Krishnan et al., 2018; Yiminniyaze et al., 2025)
Pancreatic cancer	Branched-chain amino acid metabolites	High	Pro-tumor	Plasma	Potential early diagnostic and metabolic risk biomarker (Mayers et al., 2014; Pushalkar et al., 2018)
Liver cancer	Secondary bile acids	High	Pro-tumor	Serum	Correlated with inflammation and hepatocarcinogenesis (Yoshimoto et al., 2013; Jiang et al., 2025)
Prostate cancer	Polyamines (spermidine, spermine)	High	Pro-tumor	Urine/Serum	Associated with tumor proliferation and progression (Tucker et al., 2005; Zhang et al., 2023)
Bladder cancer	Aromatic amino acid derivatives	High	Pro-tumor	Urine	Non-invasive biomarker for diagnosis and recurrence (Wigner et al., 2021; Wittmann et al., 2014)
Ovarian cancer	Tryptophan metabolites (kynurenine)	High	Pro-tumor	Serum	Linked to immune suppression and disease progression (Asangba et al., 2023; Krishnan et al., 2018)
Renal cancer	Indoxyl sulfate	High	Pro-tumor	Plasma/urine	Associated with tumor micro-environment and renal metabolic dysregulation (Bala Kumar et al., 2025; Krishnan et al., 2018)

Furthermore, dysbiosis-associated reductions in short-chain fatty acids (SCFAs), along with increased exposure to pro-inflammatory and genotoxic microbial metabolites, may further impair systemic immune dysfunction, T cell imbalance, and altered epithelial responses in the respiratory tract (Krishnan et al., 2018). Collectively, these findings underscore the potential utility of circulating microbial metabolite signatures as adjunct tools for lung cancer risk assessment and early disease detection (Yiminniyaze et al., 2025).

### 7.2 Breast cancer

Breast cancer is the most frequently diagnosed malignancy worldwide, with an estimated 2.3 million new cases and 670,000 fatalities reported in 2022; the age-standardized rate (ASR) of incidence is 47.8 per 100,000, while mortality stands at 14.5 per 100,000 (Bray et al., 2024).

The pathogenesis of breast cancer is significantly influenced by hormonal regulation, metabolic status, and systemic inflammation (Shrode et al., 2023). The involvement of the gut microbiome in breast cancer biology is partially mediated by the estrobolome, a syndicate of bacteria capable of metabolizing estrogens and estrogen conjugates (Kwa et al., 2025). In contrast to gastrointestinal cancers, metabolite effects in breast cancer are largely mediated through systemic circulation and endocrine interactions, reflecting differences in tissue-specific metabolism and immune signaling environments (Hieken et al., 2016; Krishnan et al., 2018). As outlined in Table 3, elevated levels of estrogen-like microbial metabolites in circulation can increase the bioavailability of active estrogens, thereby facilitating hormone-dependent tumor growth and proliferation (Banerjee et al., 2018).

In addition to hormonal effects, dysregulation of microbial short-chain fatty acids (SCFAs) and indole derivatives has been observed in patients with breast cancer. These metabolites affect systemic inflammation, adipose tissue immune signaling, and oxidative stress, all of which contribute to tumor promotion and progression. As a result, microbial metabolite profiles derived from serum and urine have been proposed to become promising supplementary potential biomarkers for the classification of breast cancer subtypes, risk identification, and therapy response tracking (Shrode et al., 2023).

### 7.3 Colorectal cancer

In 2022, colorectal cancer (CRC) was responsible for approximately 1.93 million cases and 930,000 deaths globally, with an age-standardized incidence rate (ASR) of 23.5 per 100,000 and a mortality rate of 11.5 per 100,000 deaths (Bray et al., 2024). The most studied cancer model in microbiome-metabolite research is colorectal cancer (CRC) due to its physical proximity to the gut microbiota (Pleguezuelos-Manzano et al., 2020).

A distinctive metabolomic signature of CRC includes diminished levels of butyrate, a tumor-suppressive short-chain fatty acid (SCFA), and increased levels of secondary bile acids, such as deoxycholic acid (DCA) (Table 3). The reduction in butyrate-producing bacteria, such as *Faecalibacterium prausnitzii* and *Roseburia* spp., impairs epithelial barrier function, reduces anti-inflammatory signaling, and decreases apoptosis induction in colonocytes (Donohoe et al., 2012). These effects are particularly pronounced in colorectal cancer due to the direct proximity of tumor cells to the gut microbiota, resulting in higher local concentrations of microbial metabolites and a uniquely shaped tumor microenvironment (Yoshimoto et al., 2013; Mima et al., 2016).

However, increased microbial conversion of primary bile acids to secondary bile acids promotes DNA damage, oxidative stress, and the activation of pro-tumorigenic pathways such as EGFR signaling, Wnt/β-catenin, and NF-κB (Yoshimoto et al., 2013). These metabolite-driven changes create a CRC-specific molecular fingerprint detectable in stool and serum, highlighting their potential clinical utility for early detection and monitoring of disease progression (Wirbel et al., 2019; Pleguezuelos-Manzano et al., 2020).

### 7.4 Prostate cancer

In 2022, prostate cancer accounted for approximately 1.47 million new cases and 397,000 deaths worldwide, with an age-standardized incidence rate (ASR) of 29.4 per 100,000 and a mortality rate of 7.3 per 100,000 individuals (Bray et al., 2024). A significant metabolic characteristic of prostate cancer is altered polyamine metabolism (Zhang et al., 2023).

As indicated in Table 3, elevated levels of spermidine and spermine have been consistently observed (Tucker et al., 2005). These metabolites facilitate rapid cell proliferation, chromatin stabilization, and tumor progression (Zhang et al., 2023). Gut microbial dysbiosis may contribute to systemic polyamine dysregulation, indirectly affecting prostate tumor biology. Microbiome-derived metabolites, including polyamines, can enter systemic circulation through intestinal absorption and reach distant organs such as the prostate. These metabolites exert their effects through receptor-mediated mechanisms and metabolic signaling pathways, influencing cellular proliferation, immune responses, and tumor progression (Byndloss et al., 2017; Krishnan et al., 2018).

Additionally, the gut microbiota modulates androgen metabolism and systemic inflammation, both of which are central to prostate cancer pathogenesis (Tucker et al., 2005). Urinary profiling of polyamines and related microbial metabolites offers a promising non-invasive approach for the diagnosis and monitoring of prostate cancer (Liu et al., 2023).

However, while systemic associations have been reported, the precise molecular mechanisms and organ-specific targets involved in prostate cancer remain incompletely understood and require further investigation (Riquelme et al., 2019).

### 7.5 Liver cancer

In 2022, primary liver cancer, primarily hepatocellular carcinoma (HCC), accounted for approximately 910,000 new cases and 830,000 deaths globally, with an age-standardized incidence rate (ASR) of 9.5 per 100,000 and a mortality rate of 8.7 per 100,000 individuals (Bray et al., 2024).

The gut-liver axis is integral to hepatocarcinogenesis, and gut microbial dysbiosis-induced overproduction of secondary bile acids, as detailed in Table 3, results in hepatocyte DNA damage, oxidative stress, and chronic inflammatory signaling pathways (Yoshimoto et al., 2013). These effects are aggravated by increased translocation of microbial endotoxins, such as lipopolysaccharides (LPS), through the portal circulation (Venkatesh et al., 2014).

Alterations in bile acid metabolism, along with disruptions in microbial lipid and amino acid metabolism, modify the hepatic tumor microenvironment, contributing to fibrotic remodeling and malignant transformation (Yoshimoto et al., 2013). These findings suggest that bile acid derivatives serve as mechanistic mediators and have been proposed as potential circulating biomarkers for liver cancer progression (Jiang et al., 2025).

### 7.6 Bladder cancer

In 2022, bladder cancer was responsible for approximately 614,000 new cases and 220,000 deaths globally, with an age-standardized incidence rate (ASR) of 5.6 per 100,000 and a mortality rate of 1.8 per 100,000 (Bray et al., 2024).

Metabolites derived from the gut microbiota and excreted through the renal system play a significant role in the urinary metabolome. As indicated in Table 3, increased levels of aromatic amino acid derivatives have been associated with bladder cancer. These metabolites may provoke chronic urothelial inflammation and oxidative DNA damage, thereby facilitating tumor initiation (Wigner et al., 2021).

Urinary metabolomics offers a highly accessible and non-invasive approach for identifying cancer-associated metabolite signatures, positioning gut-derived metabolites as promising candidates for the diagnosis and monitoring of bladder cancer recurrence (Wittmann et al., 2014).

### 7.7 Ovarian cancer

In 2022, ovarian cancer accounted for approximately 314,000 cases and 207,000 deaths globally, characterized by a high mortality-to-incidence ratio due to advanced-stage diagnoses (Bray et al., 2024). As shown in Table 3, increased levels of tryptophan metabolites, such as kynurenine, have been associated with ovarian cancer (Asangba et al., 2023). Activation of the kynurenine pathway contributes to immune suppression by inhibiting cytotoxic T-cell responses and promoting regulatory immune phenotypes (Krishnan et al., 2018).

These metabolic changes may facilitate tumor immune evasion and disease progression. Emerging serum metabolomic studies suggest that microbial tryptophan metabolites may serve as promising biomarkers for the early detection and therapy monitoring of ovarian cancer (Asangba et al., 2023).

### 7.8 Pancreatic cancer

In 2022, pancreatic cancer was responsible for approximately 511,000 new cases and 467,000 deaths globally, underscoring its notably poor prognosis and aggressiveness (Bray et al., 2024). A significant metabolic characteristic of pancreatic cancer is the increased levels of branched-chain amino acid (BCAA) metabolites, as shown in Table 3. These metabolites are associated with metabolic reprograming, insulin resistance, and systemic inflammation, all of which contribute to pancreatic tumorigenesis (Mayers et al., 2014).

The gut microbiota influences systemic BCAA levels and metabolic homeostasis, linking dysbiosis to pancreatic cancer risk (Pushalkar et al., 2018). Microbiome-derived metabolites can enter systemic circulation through intestinal absorption and portal transport, enabling their distribution to distant organs such as the pancreas. At the molecular level, these metabolites act through receptors including G-protein coupled receptors (GPR41 and GPR43), aryl hydrocarbon receptor (AhR), and other metabolic signaling pathways, thereby modulating inflammation, immune responses, and tumor-associated metabolic processes (Zelante et al., 2013; Krishnan et al., 2018). Additionally, intra-tumoral microbiota have been implicated in modulating chemo-resistance and immune evasion, highlighting the critical role of microbiome–metabolite interactions in pancreatic cancer progression. Nevertheless, although these systemic interactions are increasingly recognized, the precise organ-specific transport mechanisms and molecular targets remain to be fully elucidated (Riquelme et al., 2019).

### 7.9 Renal cancer

In 2022, renal cancer accounted for approximately 400,000 new cases and 175,000 deaths globally, with an age-standardized incidence rate (ASR) of 4.4 per 100,000 and a mortality rate of 1.5 per 100,000 individuals (Bray et al., 2024).

Recent evidence underscores the significance of the gut–kidney axis in renal cell carcinoma (RCC). As shown in Table 3, elevated levels of indoxyl sulfate, a gut-derived uremic toxin produced through microbial tryptophan metabolism, are increasingly linked to the remodeling of the renal tumor microenvironment. Mechanistically, indoxyl sulfate is transported from the circulation into renal proximal tubular cells through organic anion transporters OAT1 (SLC22A6) and OAT3 (SLC22A8), facilitating its intracellular accumulation within the renal microenvironment. Once internalized, it activates aryl hydrocarbon receptor (AhR) signaling and promotes oxidative stress-mediated pathways, leading to pro-inflammatory gene expression, endothelial dysfunction, and metabolic reprograming that collectively contribute to a tumor-permissive microenvironment (Krishnan et al., 2018; Bala Kumar et al., 2025).

However, while these mechanisms are supported by experimental and disease-associated studies, their direct contribution to renal tumorigenesis remains to be fully validated in cancer-specific models (Bala Kumar et al., 2025).

Furthermore, microbial metabolites influence responses to immune checkpoint inhibitors, with beneficial taxa, such as *Akkermansia muciniphila*, being associated with improved outcomes in RCC immunotherapy (Gopalakrishnan et al., 2018). These observations underscore that while microbiome-derived metabolites share common functional roles, their biological impact varies significantly across cancer types due to differences in tissue-specific metabolic pathways, immune landscapes, and exposure dynamics (Riquelme et al., 2019; Salgia et al., 2020).

## 8 Microbial metabolites as emerging biomarkers in cancer diagnostics

**FIGURE 4:**
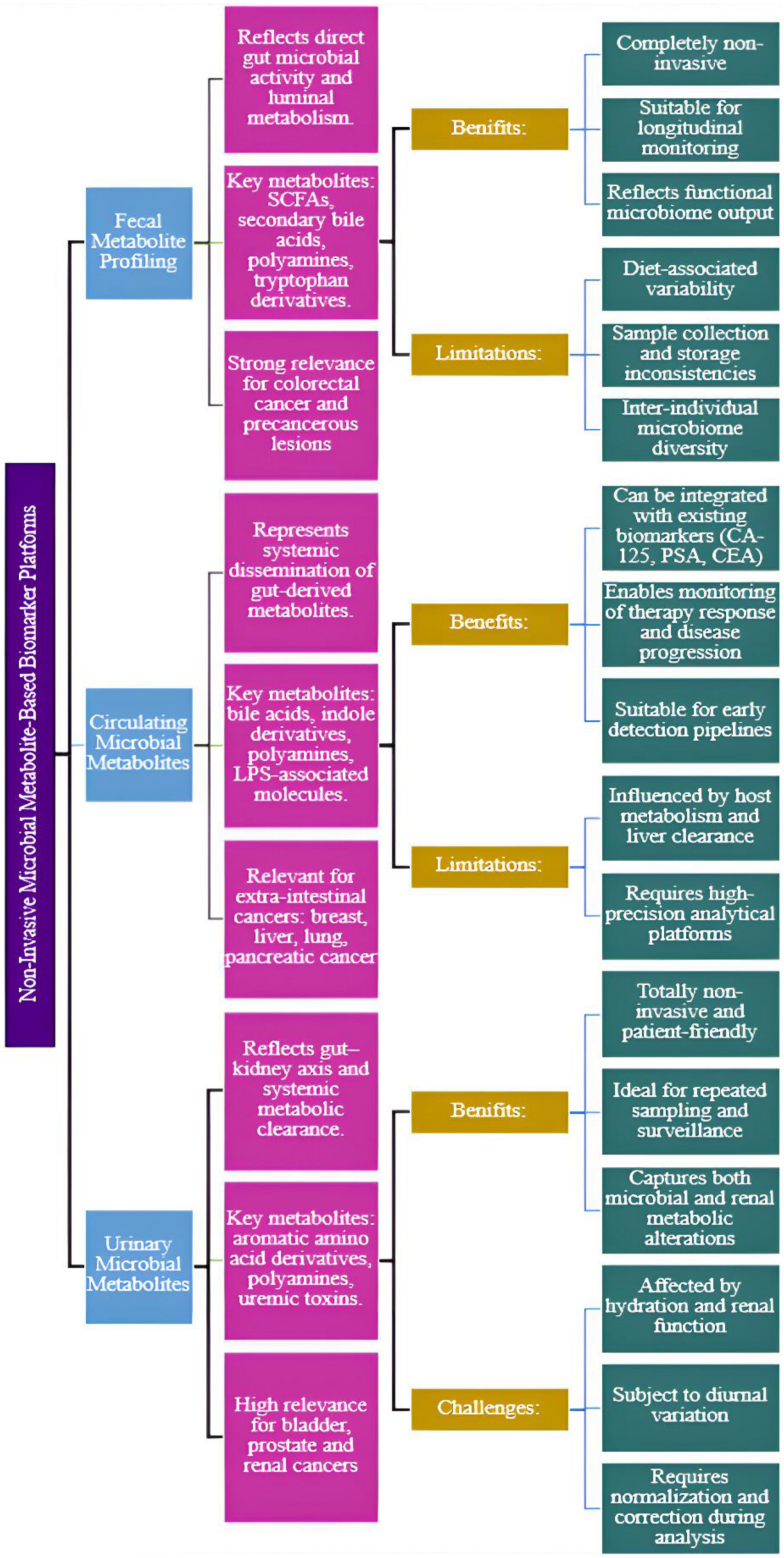
A schematic representation of non-invasive biofluid platforms for metabolite-based cancer biomarker discovery: This schematic illustrates three major non-invasive platforms for microbiome-derived metabolite profiling: fecal, circulating blood, and urinary metabolites. Fecal metabolite profiling reflects direct gut microbial activity and luminal metabolism, capturing key metabolites such as short-chain fatty acids, secondary bile acids, polyamines, and tryptophan derivatives, with strong relevance to colorectal cancer and precancerous conditions. Circulating blood microbial metabolites represent systemic dissemination of gut-derived metabolites and are associated with extra-intestinal malignancies, including breast, liver, lung, and pancreatic cancers. Urinary microbial metabolites reflect the gut-kidney axis and systemic metabolic clearance, with relevance to bladder, prostate, and renal cancers. Each platform presents distinct advantages, including non-invasiveness, suitability for longitudinal monitoring, and integration with existing biomarkers, as well as limitations such as inter-individual variability, dietary influences, and analytical constraints. Collectively, these platforms provide complementary approaches for cancer detection, monitoring, and precision diagnostics.

Microbiome-derived metabolites represent functional readouts of host-microbe interactions and therefore offer promising candidates for cancer biomarker development. Unlike microbial DNA profiling, which primarily reflects taxonomic composition, metabolite analysis captures the functional output of the microbiome and its interaction with diet, immunity, and host metabolic processes (Lozenov et al., 2023). Many of these metabolites can be quantified in accessible biological specimens, including stool, blood, and urine (Jobard et al., 2021). Through established analytical platforms, they offer opportunities for non-invasive cancer detection and risk stratification, although further validation is required (Song et al., 2025). This section underscores the predictive significance of microbial metabolites in oncology, their evolution into non-invasive biomarker platforms, and the critical methodological challenges associated with their clinical application. The role of gut microbiota-derived metabolites as molecular links between dysbiosis and cancer, and their detection in non-invasive biofluids, is illustrated in Figure 4.

### 8.1 Microbial metabolites as predictive indicators in early oncological screening

Although early cancer detection substantially improves patient outcomes, current screening strategies remain limited by invasiveness, cost, and demographic applicability (O’Donnell et al., 2017). In this context, microbial metabolites have been investigated as potential predictive biomarkers, as altered concentrations are frequently observed during early or pre-malignant stages and may precede clinical or radiological diagnosis (Jobard et al., 2021).

In colorectal cancer, shifts in stool and serum metabolites, including reduced SCFAs, elevated secondary bile acids, and increased polyamines, have been reported in adenomatous and early stage lesions (Lozenov et al., 2023). Comparable metabolomic alterations have been described in breast, liver, and pancreatic cancers (Song et al., 2022). These findings suggest that microbial metabolite profiles may reflect early tumor-associated metabolic reprograming across multiple malignancies.

Given their dynamic responsiveness to inflammation, immune activity, and metabolic stress, microbial metabolites may provide insight into transitional disease states (Song et al., 2022, 2025). When combined with artificial intelligence (AI) and multi-omics approaches, metabolite panels have been explored for population-level risk stratification; however, robust longitudinal validation remains necessary before clinical implementation can be considered (Song et al., 2025).

### 8.2 Non-invasive metabolite-based biomarker platforms

One of the primary clinical benefits of microbial metabolite biomarkers is their ability to be detected in non-invasive or minimally invasive biological samples. Various biofluids offer distinct yet complementary metabolic data, facilitating multidimensional evaluations of cancer-associated microbial metabolism (Wittmann et al., 2014; Jobard et al., 2021; Lozenov et al., 2023).

#### 8.2.1 Fecal metabolite profiles for non-invasive cancer detection

Stool samples provide direct insight into gut microbial activity and serve as a primary matrix for examining microbiome-derived metabolites, particularly in the context of gastrointestinal cancers. Fecal metabolomic panels have been reported to differentiate patients with colorectal cancer from individuals with advanced adenomas and healthy controls by profiling metabolites such as short-chain fatty acids (SCFAs), secondary bile acids, polyamines, and tryptophan derivatives. The primary advantages of fecal metabolomics are its non-invasive nature, suitability for repeated sampling, and strong representation of luminal microbial function. However, methodological challenges persist, including variability arising from diet, sample handling, storage conditions, and interindividual differences in microbiome diversity (Jobard et al., 2021; Lozenov et al., 2023). Addressing these factors is essential for achieving clinical standardization.

#### 8.2.2 Circulating microbial metabolites in oncology

Circulating metabolites in the serum and plasma reflect the systemic influence of gut-derived microbial products on distant organs. These metabolites include bile acids, indole derivatives, polyamines, lipopolysaccharide-associated molecules, and microbially modified amino acids (Jobard et al., 2021). Given that blood-based assays are already integrated into routine clinical practice, circulating microbial metabolites present a highly feasible diagnostic tool. Their integration with established clinical biomarkers such as CA-125, PSA, or CEA has been proposed as a strategy to potentially improve diagnostic performance, although further validation is required. Moreover, longitudinal measurement of circulating metabolites enables tracking of disease progression, therapeutic response, and early relapse (Song et al., 2025).

#### 8.2.3 Urine-based microbial metabolites as non-invasive readouts

Urinary metabolomics offers a non-invasive approach for monitoring microbial-host metabolic interactions, particularly in the context of urological and systemic cancers. Numerous metabolites derived from the gut are filtered by the kidneys and subsequently excreted in the urine, including aromatic amino acid derivatives, polyamines, short organic acids, and uremic toxins (Jobard et al., 2021). Urinary metabolite profiling has been reported to demonstrate potential utility in studies of bladder and prostate cancer, facilitating the differentiation between cancer patients and healthy controls. The longitudinal collection of urine samples has practical advantages for routine monitoring (Song et al., 2025). However, the interpretation of metabolites must account for factors such as hydration status, renal function, and diurnal variation to ensure reproducibility (Wittmann et al., 2014).

**FIGURE 5:**
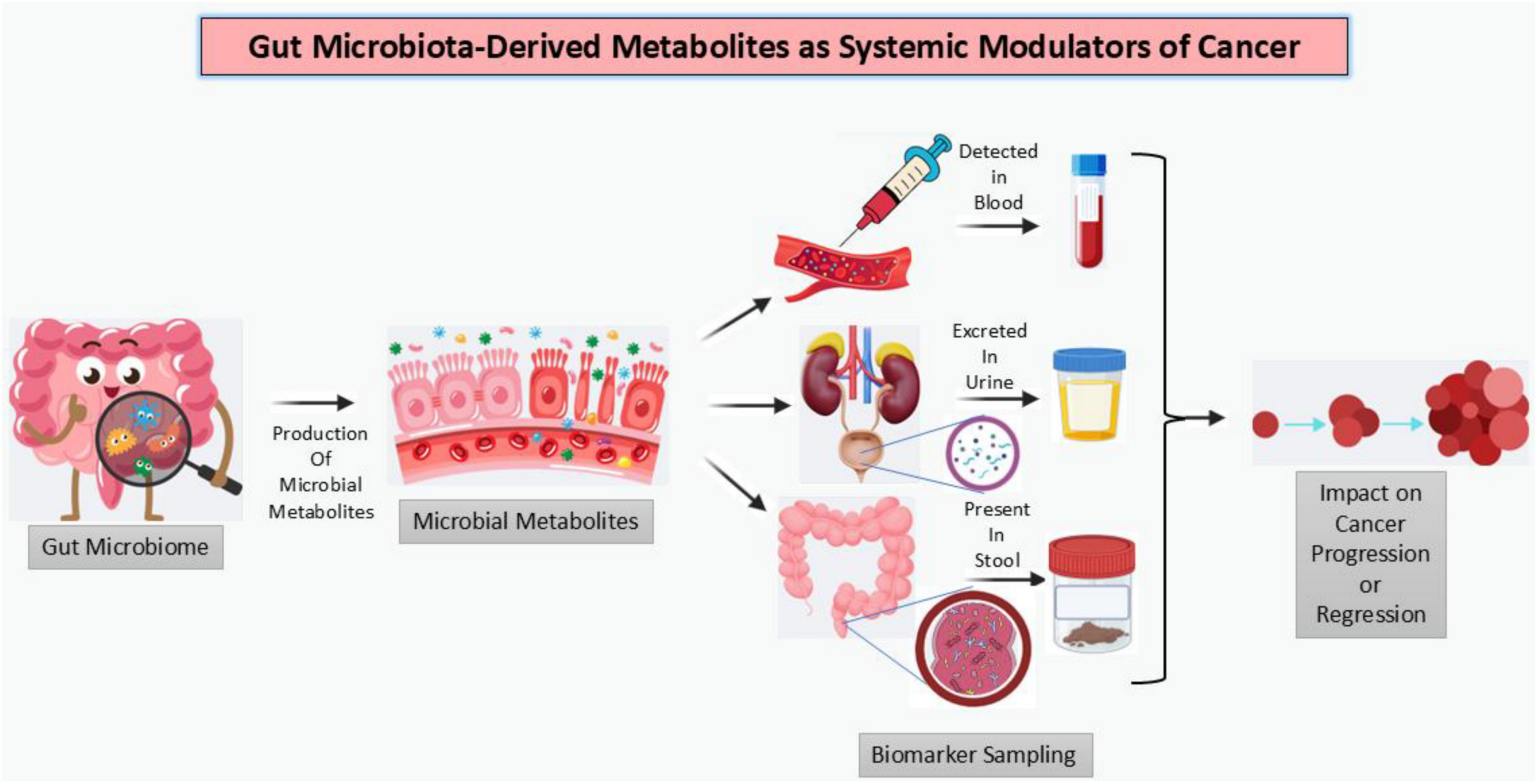
Microbiome-derived metabolites as non-invasive cancer biomarkers: Microbial metabolites produced in the gut enter systemic circulation and are detectable in biofluids such as stool, blood, and urine. These metabolites reflect functional microbiome activity and influence tumor progression or suppression, highlighting their potential as minimally invasive biomarkers for cancer diagnosis, prognosis, and therapeutic monitoring.

Figure 5 schematically illustrates the principal sample matrices employed for profiling gut microbial metabolites in oncology. Fecal samples are particularly informative for colorectal and gastrointestinal cancers because they capture luminal microbial activity (Lozenov et al., 2023). Serum and plasma metabolites, which reflect the systemic dissemination of gut-derived compounds, are clinically compatible biomarkers for extra-intestinal malignancies (Jobard et al., 2021). Urine, as a complementary matrix, captures gut-kidney metabolic interactions and is especially pertinent to bladder, prostate, and renal cancer. Collectively, these biofluids facilitate multidimensional, non-invasive evaluation of cancer-associated microbial metabolism (Song et al., 2025).

### 8.3 Methodological and translational barriers in biomarker standardization

Despite increasing interest in microbial metabolites as cancer biomarkers, several methodological and conceptual challenges currently limit their clinical translation. A primary concern is the substantial heterogeneity across studies. Variability in sample collection, storage conditions, metabolite extraction procedures, analytical platforms, and data normalization strategies contributes to inter-study inconsistency and impairs reproducibility (Wittmann et al., 2014; Wirbel et al., 2019).

Population-level variability further complicates interpretation. Diet, geographic location, ethnicity, host genetics, medication exposure, and comorbid conditions significantly influence microbiome composition and metabolic output, limiting the generalizability of proposed metabolite signatures. Many available studies are also constrained by relatively small sample sizes and single-center designs, increasing the risk of statistical overfitting and reducing external validation capacity (Wirbel et al., 2019; Jobard et al., 2021).

An additional limitation involves distinguishing causation from correlation. While altered metabolite profiles are frequently associated with malignancy, it remains unclear whether these metabolites actively contribute to carcinogenesis or reflect secondary metabolic consequences of tumor development (Wirbel et al., 2019). Longitudinal studies and mechanistic validation models are required to clarify these relationships.

Finally, successful integration of metabolite-based biomarkers into routine clinical workflows will require rigorous assessment of analytical robustness, scalability, cost-effectiveness, and regulatory approval pathways. Large-scale, multicenter validation and harmonized reporting standards will be essential before widespread clinical adoption can be achieved (Song et al., 2025).

## 9 Therapeutic targeting of microbial metabolites in cancer: current strategies and future directions

New approaches to cancer intervention have been made possible by increasing knowledge that gut microbial metabolites act as active regulators of tumor initiation, development, and therapeutic responsiveness. Modern research has shown that metabolites are modulators of immunological signaling, inflammatory pathways, epigenetic states, and medication efficacy rather than just byproducts of microbial metabolism (Furusawa et al., 2013; Krishnan et al., 2018). By therapeutically modifying these metabolites or the microorganisms that generate them, it is possible to improve traditional cancer treatment and develop personalized microbially informed oncology (Gopalakrishnan et al., 2018; Derosa et al., 2022). The subsequent subsections explore major therapeutic strategies, including dietary modulation, microbial supplementation, cutting-edge synthetic biology, and metabolite-guided precision oncology.

### 9.1 Therapeutic modulation of the gut microbiome through prebiotic, probiotic, and postbiotic interventions

One of the most direct therapeutic strategies involves modulating the gut microbiome to restore beneficial metabolite profiles (Okumura et al., 2021). Each intervention operates through distinct mechanisms: prebiotics, probiotics, and postbiotics. Collectively, these interventions constitute the foundational layer of microbiome-directed therapy, with growing interest in combination strategies that synergize with chemotherapy, radiotherapy, and immunotherapy (Sivan et al., 2015; Derosa et al., 2022).

#### 9.1.1 Prebiotics: enhancing beneficial metabolite production

Prebiotics, which are typically fermentable fibers, resistant starches, or oligosaccharides, selectively enrich commensal taxa that produce metabolites with anti-inflammatory and anti-tumorigenic properties. Prebiotics enhance epithelial barrier function, boost mucosal immunity, and mitigate systemic inflammation by promoting the growth of short-chain fatty acid (SCFA)-producing bacteria such as *Faecalibacterium, Roseburia*, and *Bifidobacterium* (Louis et al., 2010; Furusawa et al., 2013). For instance, increased butyrate availability has been associated with enhanced apoptosis in tumor cells and improved immune surveillance (Furusawa et al., 2013; Pant et al., 2017).

#### 9.1.2 Probiotics: replenishing protective microbial functions

Probiotics are live microorganisms that can restore impaired microbial function in dysbiotic gut ecosystems. Specific strains, such as *Lactobacillus reuteri, Bifidobacterium longum*, and *Akkermansia muciniphila*, have been shown to modulate cytokine responses, enhance barrier integrity, and alter metabolite profiles that support anti-tumor immunity (Everard et al., 2013; Derosa et al., 2022). In oncology, probiotics are being investigated not only for their potential to support gut health during chemotherapy by mitigating conditions such as mucositis or diarrhea but also for their capacity to enhance immunotherapy responses through the recalibration of inflammatory and metabolic pathways (Sivan et al., 2015; Derosa et al., 2022).

#### 9.1.3 Postbiotics: direct delivery of beneficial metabolites

Postbiotics, which are defined as purified microbial metabolites, structural components, or inactivated microbial preparations, offer therapeutic benefits without the safety concerns associated with live organisms. Supplementation with compounds such as butyrate, propionate, indole-3 derivatives, and microbial peptides can directly modulate immune tolerance, facilitate epithelial repair, and suppress local tumor activity (Furusawa et al., 2013; Okumura et al., 2021). This strategy circumvents the unpredictable colonization patterns of probiotics and permits the precise dosing of biologically active compounds (Zhu et al., 2025).

### 9.2 Synthetic biology and engineered microbiome strategies in precision oncology

Recent advancements in synthetic biology have revolutionized microbiome research, enabling the microbiome to function as a programmable therapeutic system rather than a passive ecological entity (Isabella et al., 2018). Engineered microorganisms now present promising opportunities for delivering therapeutic metabolites, anticancer agents, or immunomodulatory signals directly within the host organism (Danino et al., 2015; Chowdhury et al., 2019).

#### 9.2.1 Engineered bacteria for tumor-targeted delivery

Engineered bacterial strains can be developed to selectively colonize tumor microenvironments, particularly those that are hypoxic or immunosuppressed, and locally release therapeutic payloads (Zheng et al., 2017). These payloads may consist of cytotoxic molecules, short-chain fatty acids (SCFAs), pro-apoptotic compounds, immunostimulatory metabolites, or enzymes that convert prodrugs into active agents (Gurbatri et al., 2020). The localized delivery of these agents reduces systemic toxicity and enables sustained therapeutic activity within the tumor (Chowdhury et al., 2019).

#### 9.2.2 Microbial syndicates with defined functional outputs

Synthetic associations, comprising multiple microbial species with specific metabolic functions, facilitate precise modulation of ecosystems. These associations have the potential to restore disrupted metabolic pathways, stabilize dysbiotic networks, and generate metabolite combinations designed to inhibit tumor growth or enhance immune function (Sheth et al., 2016; Venturelli et al., 2018).

#### 9.2.3 Metabolite-driven gene circuitry

Advanced gene circuits enable engineered microbes to detect tumor-associated metabolites or microenvironmental signals and respond by initiating therapeutic outputs. This integration of tumor detection with targeted metabolite release represents an innovative approach in precision oncology (Chowdhury et al., 2019). Although most synthetic biology platforms are currently in experimental or early translational phases, their potential for targeted, modular, and programmable therapy positions them as one of the most promising future directions in onco-microbiome research (Riglar et al., 2017).

### 9.3 Integrating microbiome signatures into precision oncology

The variability in microbial composition and metabolic output among individuals offers significant potential for personalized oncology. Microbial metabolites are increasingly acknowledged as crucial determinants of chemotherapy efficacy, immunotherapy responsiveness, and toxicity profiles (Iida et al., 2013; Gopalakrishnan et al., 2018; Routy et al., 2018).

#### 9.3.1 Predicting therapeutic response

Distinct microbial metabolites exert significant influence on drug metabolism, T-cell activation, inflammatory thresholds, and epithelial repair processes (Iida et al., 2013). For instance, elevated levels of short-chain fatty acids (SCFAs) are associated with enhanced responses to immune checkpoint inhibitors, whereas increased concentrations of polyamines and secondary bile acids may contribute to resistance against therapy (Gopalakrishnan et al., 2018; Routy et al., 2018). Consequently, pre-treatment profiling of microbial metabolite signatures may serve as a predictive tool for assessing responses to targeted therapies, immunotherapies, or radiotherapy (Routy et al., 2018; Riquelme et al., 2019).

#### 9.3.2 Personalized intervention pathways

The integration of microbial metabolomic data with clinical, genomic, and immunological information has the potential to advance patient-specific treatment strategies (Gopalakrishnan et al., 2018; Routy et al., 2018). Future frameworks in precision oncology may advocate for personalized interventions, such as prebiotic or probiotic supplementation, engineered microbial therapies, dietary modifications, or metabolite supplementation, to enhance therapeutic outcomes while minimizing toxicity (Viaud et al., 2013; Isabella et al., 2018; Routy et al., 2018).

## 10 Challenges and future directions in onco-microbiome research

As microbiome and microbial metabolite research advances toward clinical application in oncology, it introduces a distinct set of ethical, regulatory, and translational challenges that extend beyond conventional drug development. Microbiome-based approaches involve living microbial ecosystems, generate high-dimensional biological and metabolic data, and often rely on longitudinal sample collection and reuse, raising critical concerns related to informed consent, data privacy, governance, and clinical oversight (Rhodes, 2016; Franzosa et al., 2019). Large-scale metagenomic and metabolomic studies have reported that microbial composition and metabolic output are closely associated with disease states and inflammatory phenotypes, highlighting both the diagnostic potential and ethical sensitivity of microbiome-derived data (Jobard et al., 2021).

From a regulatory perspective, translating microbiome-derived diagnostics and therapeutics into oncology practice remains challenging, as existing approval pathways were not designed to evaluate biologically active microbial products or metabolite-guided diagnostic platforms (Ott et al., 2017). Standardized procedures for sample collection and analysis, as well as thorough validation of analytical performance, clinical value, and reproducibility, are required by regulatory frameworks (Yachida et al., 2019; Jobard et al., 2021). These difficulties are especially noticeable for microbiome-based treatments, such as probiotics and modified microbial strains, where risk factors like horizontal gene transfer and genomic stability are major regulatory concerns (Hidalgo-Cantabrana et al., 2019).

Beyond ethical and regulatory issues, the global translation of microbiome-oncology research is limited by disparities in research infrastructure, regulatory standards, and population representation. Substantial variation in microbiome composition across geographic regions, ethnicities, diets, and lifestyles underscores the need for inclusive study designs and harmonized international frameworks (Wirbel et al., 2019, 2021). Future research should also prioritize integrating host-specific variables, including immune profiling, genetic variability, and metabolic status, to better understand the bidirectional nature of host-microbiome interactions in cancer (Thaiss et al., 2016; Byndloss et al., 2017). In addition to these challenges, establishing causal relationships between microbiome-derived metabolites and tumorigenesis remains a major scientific barrier. While current evidence is largely associative, future research must incorporate longitudinal cohort studies, germ-free and gnotobiotic animal models, and targeted metabolite intervention experiments to enable rigorous causal inference and mechanistic validation (Byndloss et al., 2017; Franzosa et al., 2019).

Therefore, to guarantee the fair, repeatable, and responsible integration of microbiome-based tools into clinical oncology, coordinated worldwide efforts are necessary, including shared microbiome databases and supportive established reporting requirements (Thomas et al., 2019; Wirbel et al., 2021).

### 10.1 Ethical and regulatory considerations in microbiome biobanking and data privacy

The collection and long-term storage of human microbiome samples, including stool, blood, and tissue samples, are becoming increasingly common in cancer research (Rhodes, 2016). These samples contain not only microbial data but also host genetic, metabolic, and health-related information, making them highly sensitive (Franzosa et al., 2019).

A major ethical concern is safeguarding the privacy of the participants. Microbiome profiles can potentially reveal information about an individual’s health status, diet, lifestyle, geographic origin, and disease susceptibility (Rhodes, 2016; Franzosa et al., 2019). Therefore, strict measures must be implemented to ensure secure storage, anonymization, and controlled access to microbiome and metabolomic data.

Obtaining informed consent is equally critical. Participants should be clearly informed about how their samples and data will be used, how long they will be stored, whether they may be shared with other researchers, and whether they may be used for future studies beyond the original study scope. Transparent biobanking practices are essential for maintaining public trust and enabling sustainable microbiome research (Rhodes, 2016).

### 10.2 Regulatory frameworks for microbiome-based therapeutics

Translating discoveries related to the microbiome and metabolites into clinical applications presents distinct regulatory challenges. Traditional frameworks for drug and diagnostic approval were not initially designed to accommodate living organisms or biologically active microbial products (Ott et al., 2017). In the context of microbiome-based diagnostics, such as metabolite-derived biomarker panels, regulatory bodies necessitate rigorous validation of analytical accuracy, clinical sensitivity, specificity, reproducibility, and clinical utility (Jobard et al., 2021).

Furthermore, standardizing sample collection, processing, and analytical methodologies is crucial to ensure consistency across clinical environments (Yachida et al., 2019). For microbiome-based therapeutics, including probiotics, engineered microbes, and postbiotic formulations, the evaluation of safety becomes even more paramount. Experimental studies evaluating genomic stability and horizontal gene transfer (HGT) in probiotic strains provide essential safety data for live microbial therapeutics. These studies are reported to demonstrate that the presence of mobile genetic elements and strain-specific defense mechanisms can significantly affect the probability of HGT. This is a major regulatory concern because gene transfer events may modify microbial colonization patterns, metabolic functions, and interactions between the microbe and the host (Hidalgo-Cantabrana et al., 2019).

### 10.3 Bridging global disparities in microbiome-oncology research

A significant impediment to the global integration of microbiome research into healthcare is the absence of harmonized standards across nations and institutions. Variations in regulatory policies, ethical guidelines, and technological infrastructure result in disparities in the collection, interpretation, and application of microbiome data (Thomas et al., 2019). The microbiome signatures exhibit considerable variation across geographic regions, ethnicities, diets, and lifestyles (Wirbel et al., 2019). Furthermore, it is imperative to ensure that microbiome research encompasses diverse populations and does not disproportionately represent data from limited demographic groups.

The global harmonization of research protocols, data-sharing standards, and clinical validation frameworks is essential for the equitable and effective application of microbiome-based cancer tools worldwide (Wirbel et al., 2021). Collaborative international initiatives, standardized reporting guidelines, and shared public microbiome databases can facilitate bridging these gaps and expedite the safe and inclusive translation of microbiome-metabolite research into clinical oncology.

### 10.4 Knowledge gaps and emerging research frontiers

Although there is an increasing body of evidence linking gut microbial metabolites to cancer biology, the field remains in a relatively nascent stage of development (Yachida et al., 2019; Jobard et al., 2021). Much of the existing knowledge is derived from associative studies, limited cohorts, and heterogeneous methodologies (Thomas et al., 2019). Identifying and addressing these gaps is essential for transitioning from descriptive microbiome research to a mechanistic understanding and clinical application. This section underscores key unresolved questions and priority areas that must be addressed to advance microbiome-metabolite research in oncology.

### 10.5 Establishing causality in microbiome-cancer interactions

One of the primary challenges in microbiome-cancer research is the differentiation between causal relationships and mere associations (Thomas et al., 2019). Many studies have found altered microbial compositions and metabolite profiles in cancer patients, but it often remains questionable if these changes are associated by tumor growth, host metabolism, or therapeutic effects, or if they directly contribute to the development of cancer (Yachida et al., 2019; Jobard et al., 2021).

The majority of available evidence is derived from cross-sectional or retrospective study designs, which are inadequate for supporting causality (Thomas et al., 2019). To investigate the tumor-modifying effects of particular microorganisms and metabolites, well-designed mechanistic studies using gnotobiotic animal models are desperately needed (Wu et al., 2009; Yoshimoto et al., 2013). The controlled regulation of microbial metabolites and host cell responses, as well as microbial depletion and reconstitution investigations for targeted metabolite supply or inhibition, are made possible by these organoid and microfluidic tumor-on-a-chip systems. They also make it easier to do temporal analysis to track changes in metabolites and microbes before the development of tumors, during their progression, and following treatment (Dijkstra et al., 2018).

Additionally, the integration of host genetics is necessary to elucidate gene-microbe-metabolite interactions. Supporting causality will also necessitate the integration of temporal data, genetic models, and functional validation studies that transcend correlation and address the biological effects of specific metabolite-host interactions at the molecular and cellular levels (Yoshimoto et al., 2013).

### 10.6 Advancing microbiome-cancer research

One significant deficiency in the field is the absence of extensive longitudinal studies. The majority of existing human research captures microbiome and metabolite profiles at a singular time point, thereby providing only a static representation of a dynamic ecosystem (Thomas et al., 2019). Cancer development, however, is a temporally dependent process, characterized by progressive alterations in host physiology, immune responses, and microbial ecology (Yachida et al., 2019).

Longitudinal cohort studies, which monitor individuals from pre-disease or early disease stages through progression and treatment, are crucial for understanding the evolution of microbial metabolites and their association with cancer risk, progression, and therapeutic outcomes (Routy et al., 2018; Yachida et al., 2019). Furthermore, multi-center and multi-population studies are necessary to ensure that findings are applicable across diverse geographic regions, dietary habits, genetic backgrounds, and environmental conditions. Without such diversity, there is a risk of developing biomarker signatures that are specific to certain populations and not universally applicable (Thomas et al., 2019; Wirbel et al., 2019).

### 10.7 Standardization and validation imperatives

A significant technical challenge in microbiome-metabolite research is the absence of standardized protocols for metabolomic analysis and multi-omics integration (Yachida et al., 2019; Jobard et al., 2021). Variations in sample collection, storage conditions, extraction methods, instrument platforms, and data-processing pipelines contribute to considerable variability across studies. This lack of harmonization complicates the comparison of findings, replication of results, and the development of unified biomarker frameworks. Establishing standardized guidelines for metabolomic workflows, reporting formats, and data normalization methods is essential for enhancing reproducibility and reliability. Comparative analyses across independent cancer cohorts have reported demonstrating that improved harmonization of metabolomic workflows enhances reproducibility and cross-study interpretability (Wirbel et al., 2019). Additionally, the integration of microbiome, metabolome, transcriptome, proteome, and clinical data remains computationally challenging (Wirbel et al., 2019, 2021; Yachida et al., 2019). More robust bioinformatics pipelines and cross-disciplinary collaborations among wet-lab scientists, clinicians, and data scientists are necessary to derive interpretable and clinically relevant outcomes from these extensive, multidimensional datasets (Wirbel et al., 2019).

### 10.8 Inclusive understanding of the gut-tumor axis across ethnicities

Current literature on the microbiome-cancer relationship predominantly originates from studies conducted within limited geographic regions, often lacking representation from diverse ethnic and socioeconomic groups (Wirbel et al., 2019). Factors such as diet, lifestyle, genetics, environmental exposures, and healthcare access significantly influence gut microbiome composition and metabolite production (Yachida et al., 2019). To develop microbiome-based cancer biomarkers and therapies that are globally applicable, it is imperative to include diverse populations and examine how cultural, dietary, and genetic variations affect the gut-tumor axis. Without such inclusivity, there is a risk of perpetuating healthcare disparities and creating tools that do not apply to underrepresented populations. Future research should prioritize inclusive study designs, foster cross-country collaborations, and conduct region-specific analyses that acknowledge the impact of sociobiological diversity on microbiome-cancer interactions (Wirbel et al., 2019).

## 11 Conclusion

In summary, this review integrates current evidence on the multifaceted role of gut microbial metabolites in cancer biology, encompassing dysbiosis-associated alterations, mechanistic signaling pathways, analytical advancements in metabolomics, cancer-specific metabolic signatures, biomarker potential, and emerging therapeutic strategies. The collective information discussed in this review indicates that microbial metabolites function as active molecular intermediates that shape tumor initiation, progression, regression, immune modulation, and therapeutic responsiveness across diverse malignancies.

Rather than representing passive byproducts of microbial activity, these metabolites reflect the dynamic interface between the host, microbiota, and environmental exposures. Through their influence on inflammatory signaling, epigenetic regulation, metabolic reprograming, genomic stability, and tumor microenvironment remodeling, microbiome-derived metabolites contribute to complex oncogenic and tumor-suppressive processes. Their detection in accessible biological matrices such as stool, blood, and urine highlights their translational relevance as candidate non-invasive biomarkers.

Across multiple cancer types, distinct metabolite patterns have been reported, suggesting disease-specific as well as shared metabolic signatures. However, despite promising associations, significant challenges remain. These include methodological heterogeneity across analytical platforms, limited longitudinal validation, population variability, and the need to distinguish causative mechanisms from secondary tumor-associated metabolic changes (Franzosa et al., 2019; Jobard et al., 2021). Addressing these limitations through standardized workflows, multi-center validation studies, and mechanistic modeling will be essential before clinical implementation can be fully realized (Thomas et al., 2019).

Importantly, the complexity of cancer cannot be adequately understood through isolated molecular layers. A systems-level perspective that integrates microbiome dynamics with host genetics, immune responses, metabolic networks, and environmental influences provides a more comprehensive framework for interpreting tumor-microbiome interactions. Future investigations combining metagenomics, metabolomics, transcriptomics, and clinical datasets may facilitate the transition from descriptive association toward predictive and mechanistic oncology models.

Rather than focusing on individual microbe-metabolite associations, this review synthesizes current evidence into a unified framework, highlighting shared mechanistic pathways and context-dependent variability across cancer types. Collectively, while microbiome-derived metabolites represent a promising and evolving dimension of cancer research, continued methodological rigor, interdisciplinary collaboration, and translational validation are necessary to responsibly advance this field toward precision oncology applications. Recognizing cancer as a biologically interconnected ecosystem shaped by host-microbe interactions may open more refined avenues for prevention, diagnosis, and therapeutic modulation.
